# A Transcriptional Signature of Fatigue Derived from Patients with Primary Sjögren’s Syndrome

**DOI:** 10.1371/journal.pone.0143970

**Published:** 2015-12-22

**Authors:** Katherine James, Shereen Al-Ali, Jessica Tarn, Simon J. Cockell, Colin S. Gillespie, Victoria Hindmarsh, James Locke, Sheryl Mitchell, Dennis Lendrem, Simon Bowman, Elizabeth Price, Colin T. Pease, Paul Emery, Peter Lanyon, John A. Hunter, Monica Gupta, Michele Bombardieri, Nurhan Sutcliffe, Costantino Pitzalis, John McLaren, Annie Cooper, Marian Regan, Ian Giles, David Isenberg, Vadivelu Saravanan, David Coady, Bhaskar Dasgupta, Neil McHugh, Steven Young-Min, Robert Moots, Nagui Gendi, Mohammed Akil, Bridget Griffiths, The UK Primary Sjögren’s Syndrome registry, Anil Wipat, Julia Newton, David E. Jones, John Isaacs, Jennifer Hallinan, Wan-Fai Ng

**Affiliations:** 1 Interdisciplinary Computing and Complex BioSystems Research Group, Newcastle University, Newcastle upon Tyne, United Kingdom; 2 Musculoskeletal Research Group, Institute of Cellular Medicine, Newcastle University, Newcastle upon Tyne, United Kingdom; 3 Department of Biology, College of Science, University of Basrah, Basrah, Iraq; 4 Bioinformatics Support Unit, Newcastle University, Newcastle upon Tyne, United Kingdom; 5 School of Mathematics & Statistics, Newcastle University, Newcastle upon Tyne, United Kingdom; 6 Newcastle-upon-Tyne Hospitals NHS Foundation Trust, Newcastle upon Tyne, United Kingdom; 7 Rheumatology Department, University Hospital Birmingham, Birmingham, United Kingdom; 8 Great Western Hospitals NHS Foundation Trust, Swindon, United Kingdom; 9 Section of Musculoskeletal Disease, Leeds Institute of Molecular Medicine, University of Leeds & NIHR Leeds Musculoskeletal Biomedical Research Unit, Leeds Teaching Hospitals Trust, Leeds, United Kingdom; 10 Nottingham University Hospital, Nottingham, United Kingdom; 11 Gartnavel General Hospital, Glasgow, United Kingdom; 12 Barts and the London NHS Trust & Barts and the London School of Medicine and Dentistry, London, United Kingdom; 13 NHS Fife, Whyteman’s Brae Hospital, Kirkcaldy, United Kingdom; 14 Royal Hampshire County Hospital, Winchester, United Kingdom; 15 Portsmouth Hospitals NHS Trust, Portsmouth, United Kingdom; 16 Royal Derby Hospital, Derby, United Kingdom; 17 University College London Hospitals NHS Foundation Trust, London, United Kingdom; 18 Queen Elizabeth Hospital, Gateshead, United Kingdom; 19 Sunderland Royal Hospital, Sunderland, United Kingdom; 20 Southend University Hospital, Southend, United Kingdom; 21 Royal National Hospital for Rheumatic Diseases, Bath, United Kingdom; 22 Aintree University Hospitals, Liverpool, United Kingdom; 23 Basildon Hospital, Basildon, United Kingdom; 24 Royal Hallamshire Hospital, Sheffield, United Kingdom; 25 Institute of Cellular Medicine, Newcastle University, Newcastle upon Tyne, United Kingdom; 26 National Institute for Health Research, Newcastle Biomedical Research Centre, Newcastle upon Tyne, United Kingdom; 27 BioThink Pty Ltd, Brisbane, Australia; Charité University Medicine Berlin, GERMANY

## Abstract

**Background:**

Fatigue is a debilitating condition with a significant impact on patients’ quality of life. Fatigue is frequently reported by patients suffering from primary Sjögren’s Syndrome (pSS), a chronic autoimmune condition characterised by dryness of the eyes and the mouth. However, although fatigue is common in pSS, it does not manifest in all sufferers, providing an excellent model with which to explore the potential underpinning biological mechanisms.

**Methods:**

Whole blood samples from 133 fully-phenotyped pSS patients stratified for the presence of fatigue, collected by the UK primary Sjögren’s Syndrome Registry, were used for whole genome microarray. The resulting data were analysed both on a gene by gene basis and using pre-defined groups of genes. Finally, gene set enrichment analysis (GSEA) was used as a feature selection technique for input into a support vector machine (SVM) classifier. Classification was assessed using area under curve (AUC) of receiver operator characteristic and standard error of Wilcoxon statistic, SE(W).

**Results:**

Although no genes were individually found to be associated with fatigue, 19 metabolic pathways were enriched in the high fatigue patient group using GSEA. Analysis revealed that these enrichments arose from the presence of a subset of 55 genes. A radial kernel SVM classifier with this subset of genes as input displayed significantly improved performance over classifiers using all pathway genes as input. The classifiers had AUCs of 0.866 (SE(W) 0.002) and 0.525 (SE(W) 0.006), respectively.

**Conclusions:**

Systematic analysis of gene expression data from pSS patients discordant for fatigue identified 55 genes which are predictive of fatigue level using SVM classification. This list represents the first step in understanding the underlying pathophysiological mechanisms of fatigue in patients with pSS.

## Introduction

Severe, debilitating fatigue is a common symptom in a wide range of chronic diseases including autoimmune diseases and cancers [1–6], and is a side effect of treatments such as chemotherapies, radiotherapies [7, 8] and some medications [9]. Fatigue is a tiredness which may be mental, physical, or both, and that results in an inability to function at normal performance levels. Chronic fatigue is a disabling symptom that is a major cause of loss of productivity and has a substantial healthcare-related cost [10, 11]. However, the underlying pathophysiological mechanisms of fatigue remain unclear and treatment of fatigue is currently largely ineffective [12].

There is a clear need to identify a biological signature of fatigue in order to advance our understanding of its pathophysiological mechanisms. Such a signature will inform therapeutic development, aid in drug target identification, and act as a biomarker to measure responses to interventions. Although the biological basis of fatigue remains unknown, recent data indicate that immune dysregulation is common among fatigued patients and may play a key role in the biological mechanisms of fatigue. Chronic fatigue is a common symptom in many conditions involving a dysregulated immune system, such as autoimmune diseases [13, 14]. IFN*α* and other cytokine therapies often induce fatigue [9]. Conversely, therapies that interfere with, or modify, cytokine signalling have been found to reduce fatigue [15].

Research suggests that severe fatigue in these diverse conditions is driven by similar biological mechanisms [16] and, therefore, a variety of diseases may be valuable as disease models for fatigue. We propose the multisystem autoimmune disease primary Sjögren’s Syndrome (pSS) as a model to investigate the biological signature of fatigue. This disease is characterised by oral and ocular dryness, profound fatigue and musculoskeletal pain [17]. The disease affects approximately 0.04% of the population, with a female to male ratio of around 9:1 [18].

There are well-established diagnostic criteria for pSS [19, 20]. Although disabling chronic fatigue is common among pSS, some suffer minimal symptoms of fatigue. This discordance in fatigue provides an opportunity to uncover biological changes associated with pSS-related fatigue by the comparison of patients with different fatigue levels. For instance, it is now established that type I IFN signature is present in the majority of, but not all, pSS patients [21], and that IFN*α* treatment can induce fatigue. It would therefore be of interest to investigate whether fatigue in pSS is associated with the presence of this IFN signature. Importantly, the correlation between fatigue and disease activity in pSS is weak, suggesting that a distinct biological process may be responsible for fatigue symptoms [22]. Furthermore, the majority of pSS patients do not receive immuno-modulatory therapies that may confound the study of fatigue-specific changes in cohort studies [23].

Here, we compare global gene expression profiles of whole blood from a group of pSS patients with differing levels of fatigue using multiple statistical and machine learning techniques. Gene set enrichment analysis identifies 55 genes which are collectively associated with fatigue. Using this gene signature a support vector machine classifier is created which is predictive of fatigue level in this group. These genes provide a potential basis for the future study of fatigue in pSS in order to develop mechanistically-informed approaches to therapy.

## Results

### Patient Characteristics

**Fig 1:**
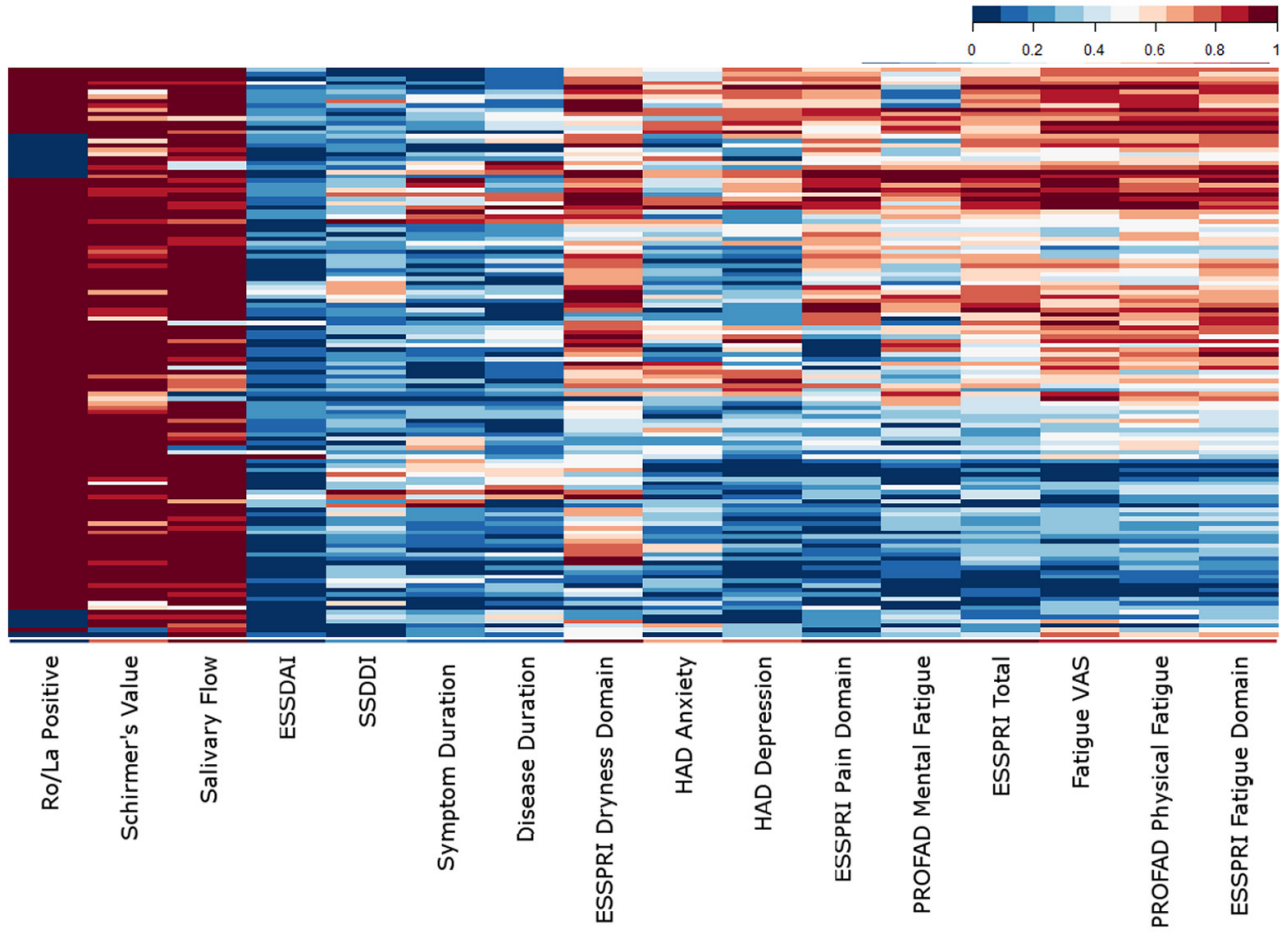
The characteristics of the patients. A heatmap of the clinical scores for the 133 patients included in this study. The values have been scaled between zero (absent) and one (worst). ESSDAI = EULAR Sjögren’s Syndrome Disease Activity Index, SSDDI = Sjögren’s Syndrome Disease Damage Index, ESSPRI = EULAR Sjögren’s Syndrome Patient Reported Index, HAD = Hospital Anxiety and Depression, PROFAD = Profile of Fatigue and Discomfort, VAS = Visual Analogue Scale.

Table 1 summarises the demographics of the subjects used in this study. The pSS patient group covered a range of fatigue levels and symptom profiles to allow analysis of fatigue as a continuous variable (Fig 1). The Fatigue VAS cutoffs (>75/<25) produced groups of 38 high fatigue and 21 low fatigue patients. Although fatigue was moderately correlated with depression and pain, there was no association with disease activity (see S1 Table).

**Table 1:** Patient and control characteristics. The demographics and symptom levels of the patients used in this study.

	**Patient**	**Control**
**Age** (years—mean, SD)	61.16±12.12	54.40±13.05
**Disease duration** (years—mean, SD)	7.38±6.29	N/A
**Symptom duration** (years—mean, SD)	13.95±10.25	N/A
**Age at onset** (years—mean, SD)	47.22±14.46	N/A
**ESSDAI** (median, IQ)	5.00, 2.00–9.00	N/A
**SSDDI** (median, IQ)	5.00, 3.00–5.00	N/A
**Fatigue VAS** (median, IQ)	55.00, 31.00–77.00	N/A
**PROFAD-Physical** (median, IQ)	3.75, 2.25–5.00	N/A
**PROFAD-Mental** (median, IQ)	3.00, 1.50–4.00	N/A
**HADS** Anxiety (median, IQ)	7.00, 4.00–10.75	N/A
**HADS** Depression(median, IQ)	5.00, 2.50–9.00	N/A
**Total ESSPRI** (median, IQ)	5.67, 3.67–7.33	N/A
**Pain** sub-domain (median, IQ)	4.00, 2.00–7.00	N/A
**Fatigue** sub domain (median, IQ)	5.00, 3.00–8.00	N/A
**Dryness** sub domain (median, IQ)	7.00, 4.00–8.00	N/A

SD = standard deviation, IQ = interquartile range, ESSDAI = EULAR Sjögren’s Syndrome Disease Activity Index, SSDDI = Sjögren’s Syndrome Disease Damage Index, ESSPRI = EULAR Sjögren’s Syndrome Patient Reported Index, HAD = Hospital Anxiety and Depression, PROFAD = Profile of Fatigue and Discomfort.

### Differential gene expression between fatigue groups

Following transformation and normalisation of the raw data, two outliers were detected by the arrayQualityMetrics package and discarded from the remainder of the analyses (see S1 Fig). Filtering for detection threshold resulted in the loss of 39.8% of the probes. The data were then batch corrected to remove non-biological effects produced by variation between experimental batches (see S2 Fig).

**Fig 2:**
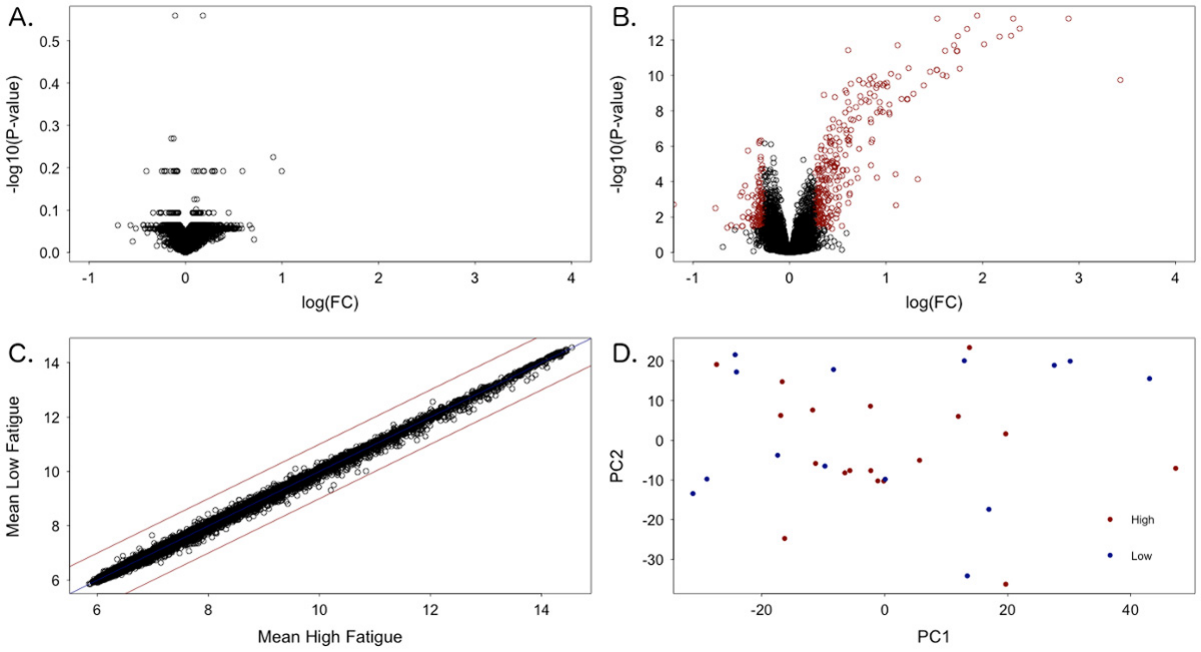
Differential gene expression analysis. (A) Volcano plot of high fatigue against low fatigue. No significant differentially expressed genes (DEGs) were detected. (B) Volcano plot of patients against healthy controls. Red points indicate DEGs with a fold change >1.2 and *p*-value <0.05. (C) The mean expression values for each gene for the high and low fatigue groups. (D) Plot of the first two principal components of the expression dataset coloured by high and low fatigue groups.

**Fig 3:**
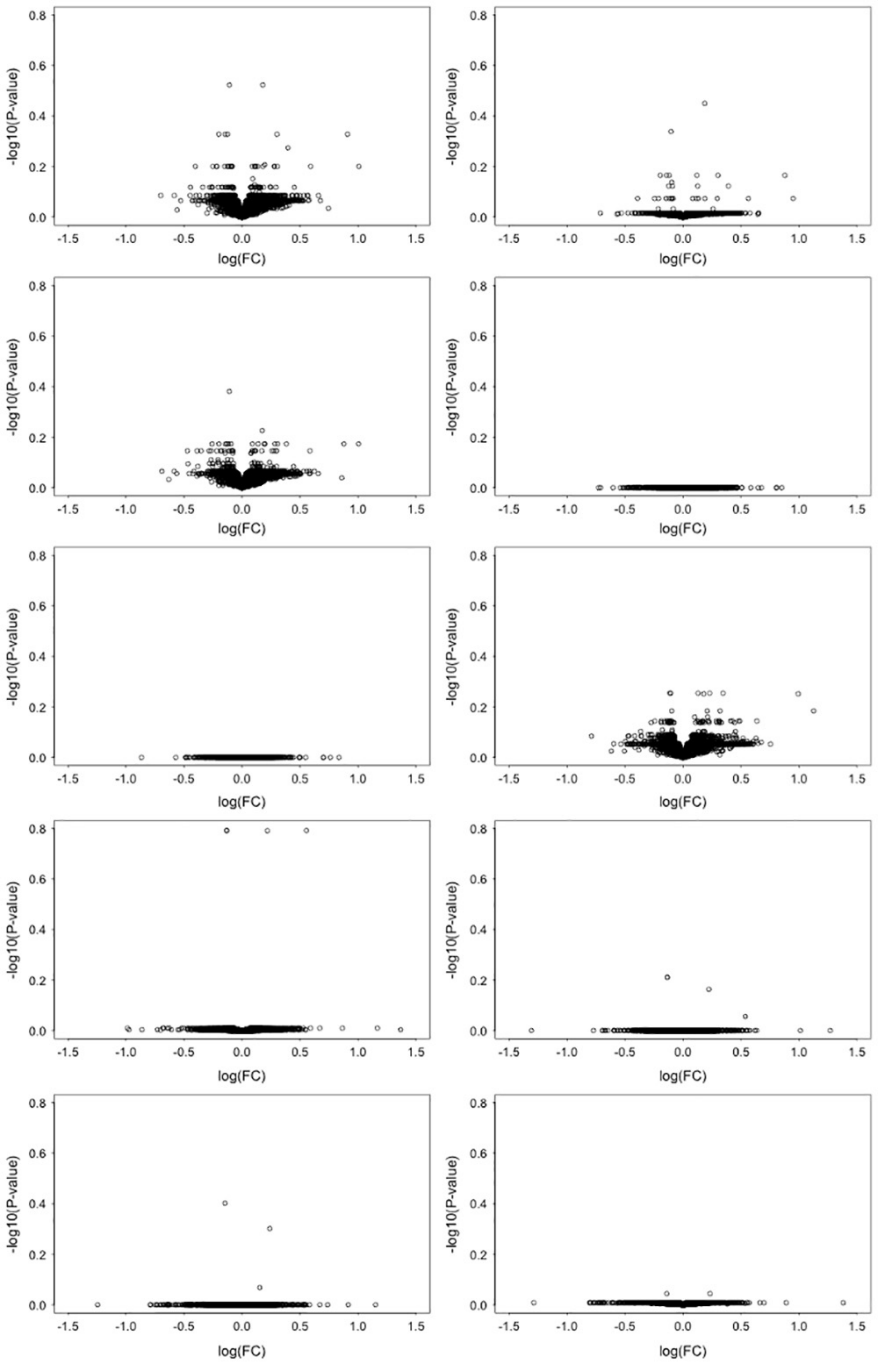
Correction for other clinical factors. Volcano plots for the Fatigue VAS fatigue groups corrected for clinical factors: (A) Age at UKPSSR cohort recruitment. (B) Disease activity measured using the EULAR Sjögren’s Syndrome Disease Activity Index. (C) Disease damage measured using the Sjögren’s Syndrome Disease Damage Index. (D) The EULAR Sjögren’s Syndrome Patient Reported Index dryness sub-domain. (E) The EULAR Sjögren’s Syndrome Patient Reported Index pain sub-domain. (F) Anxiety measured using the Hospital Anxiety and Depression scale. (G) Depression measured using the Hospital Anxiety and Depression scale. (H) Pain and depression (E & G). (I) Pain, depression, dryness and anxiety (D-G). (J) All seven factors (A-G). No significantly differentially expressed genes were identified following any correction.

Although 334 differentially expressed genes (DEGs) were detected between the pSS patients and the controls, no DEGs were detected between the high and low fatigue groups (Fig 2A and 2B). Comparison of the average expression values between the groups, and Principal Component Analysis, indicated that there was no significant difference between the fatigue groups in terms of expression (Fig 2C and 2D). When the analysis was repeated with correction for the other clinical factors, no significant DEGs were identified (Fig 3). Finally, the Fatigue VAS was analysed as continuous variables by fitting a linear regression model to the expression data. No statistically significantly DEGs were identified for any of the scores either before or after correction for other clinical variables. When these analyses were repeated using the other available fatigue scores at comparable cutoffs, no DEGs were identified in any case (see S3, S4, and S5 Figs, S2 and S3 Tables).

### Interferon type I score in fatigue groups

**Fig 4:**
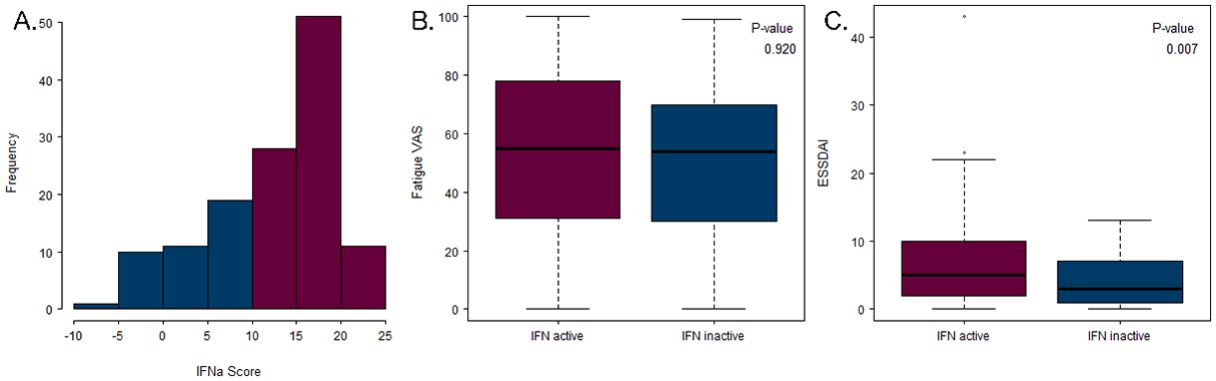
Interferon type I signature and fatigue. (A) The IFN score ranges for the 133 patients. (B) The Fatigue VAS scores for the IFN-active and IFN-inactive groups. (C) The ESSDAI scores for the IFN-active and IFN-inactive groups.

IFN activation scores ranged from −5.2 to 22.2 with a mean score of 12.5 (Fig 4A). In total, 69% of the patients (90 of 131) were IFN-active. No significant relationship was observed between IFN activation score and fatigue level (Fig 4B). Further, IFN activation was not linked to ESSPRI or SSDDI (see S6 Fig). However, ESSDAI scores were significantly higher in the IFN-positive group (Fig 4C), consistent with published data [21].

### Gene set enrichment in fatigue subsets

Gene set enrichment analysis was carried out using the Fatigue VAS high and low fatigue groups with both real and absolute gene ordering, in order to identify fatigue-related metabolic pathways. All available MSigDB C2:CP modules were tested [24], comprising canonical representations of biological pathways compiled by domain experts for the BioCarta [25], Reactome [26] and KEGG [27] databases. Three actin-related BioCarta pathways and 15 G-protein signalling Reactome pathways were found to be enriched in the high fatigue group (Table 2). Additionally, an incretin-related Reactome pathway was found to have a non-random distribution following absolute gene ordering, with enrichment split between the high and low fatigue groups. No KEGG pathways were enriched in any of the analyses. When the analysis was repeated using patients and healthy controls, 94 pathways were enriched in the pSS group (see S4 Table). One of these pathways, “Incretin synthesis, secretion, and activation” overlapped with those enriched in the high fatigue group.

**Table 2:** Enriched pathways between the Fatigue VAS high fatigue and low fatigue groups. Gene sets were considered to be enriched at an FDR cut-off of 25%. All the enriched gene sets were associated with high fatigue with the exception of incretin synthesis secretion and inactivation (*), which had a non-random distribution of enriched genes between the two fatigue groups.

**Name**	**Size**	**ES**	**NES**	**Nominal *p-* value**	**FDR *q-* value**
**BioCarta**					
CDC42RAC pathway	16	-0.798	-1.950	0	0.001
ACTINY pathway	19	-0.651	-1.848	0.002	0.007
MPR pathway	34	-0.506	-1.697	0.004	0.078
**Reactome**					
Regulation of insulin secretion by glucagon-like peptide-1	42	-0.628	-1.983	0	0.027
G beta:gamma signalling through PLC beta	20	-0.762	-1.823	0	0.052
G beta:gamma signalling through PI3Kgamma	25	-0.694	-1.793	0	0.065
Activation of kainate receptors upon glutamate binding	31	-0.629	-1.824	0	0.069
G-protein beta:gamma signalling	28	-0.691	-1.846	0	0.078
Prostacyclin signalling through prostacyclin receptor	19	-0.750	-1.762	0	0.083
Inhibition of insulin secretion by adrenaline/noradrenaline	25	-0.651	-1.697	0.002	0.113
Glucagon-type ligand receptors	33	-0.564	-1.703	0.002	0.116
G-protein activation	27	-0.669	-1.717	0.002	0.126
Thromboxane signalling through TP receptor	23	-0.698	-1.703	0	0.129
Glucagon signaling in metabolic regulation	33	-0.565	-1.664	0.002	0.155
Aquaporin-mediated transport	50	-0.548	-1.646	0	0.174
ADP signalling through P2R purinoceptor1	25	-0.658	-1.625	0.008	0.206
Thrombin signalling through proteinase activated receptors PARs	32	-0.634	-1.592	0.006	0.237
Regulation of water balance by renal aquaporins	43	-0.512	-1.595	0.002	0.247
Incretin synthesis, secretion, and inactivation*	21	0.584	1.522	0.015	0.247

ES = enrichment score, NES = normalised enrichment score, FDR = false discovery rate.

Leading edge analysis was carried out to identify the genes that contributed to the high fatigue enrichment of the BioCarta and Reactome pathways and their overlaps. This analysis indicated that the actin-related pathways had seven overlapping genes, while the G-protein signalling pathways had just five overlapping genes (Tables 3 and 4). The incretin-related pathway had five leading edge genes, LEP, DPP4, ISL1, SEC11C and SPCS1, associated with low fatigue and six genes, SPSC3, GATA4, PCSK1, GIP, FFAR1 and GCG, associated with high fatigue. There was very little overlap between the leading edges of the actin and G-protein signalling genes, or with the leading edge of the incretin-related pathway (Tables 4 and 5).

**Table 3:** Genes in the leading edge of the enriched actin-related BioCarta pathways. Genes found in leading edge overlap are shown in bold.

**Symbol**	**Name**
**ACTR2**	**ARP2 actin-related protein 2 homolog (yeast)**
**ACTR3**	**ARP3 actin-related protein 3 homolog (yeast)**
**ARPC1B**	**Actin related protein 2/3 complex, subunit 1B, 41kDa**
**ARPC2**	**Actin related protein 2/3 complex, subunit 2, 34kDa**
**ARPC3**	**Actin related protein 2/3 complex, subunit 3, 21kDa**
**ARPC4**	**ARPC4 actin related protein 2/3 complex, subunit 4, 20kDa**
**ARPC5**	**Actin related protein 2/3 complex, subunit 5, 16kDa**
CAP1	CAP, adenylate cyclase-associated protein 1 (yeast)
CDC25C	Cell division cycle 25C
CDC42	Cell division cycle 42
GNAI1	Guanine nucleotide binding protein, alpha inhibiting activity polypeptide 1
NCKAP1	NCK-associated protein 1
PAK1	p21 protein (Cdc42/Rac)-activated kinase 1
PAQR7	Progestin and adipoQ receptor family member VII
PIK3CA	Phosphatidylinositol-4,5-bisphosphate 3-kinase, catalytic subunit alpha
PIK3R1	Phosphoinositide-3-kinase, regulatory subunit 1 (alpha)
PIN1	Peptidylprolyl cis/trans isomerase, NIMA-interacting 1
PIR	Pirin (iron-binding nuclear protein)
PRKAR1A	Protein kinase, cAMP-dependent, regulatory, type I, alpha
PRKAR2A	Protein kinase, cAMP-dependent, regulatory, type II, alpha
RHOA	Ras homolog family member A
WASF2	WAS protein family, member 2
WASL	Wiskott-Aldrich syndrome-like

**Table 4:** Genes in the leading edge of the enriched Reactome G-protein signalling pathways. Genes found in leading edge overlap are shown in bold.

**Symbol**	**Name**
AQP10	Aquaporin 10
AQP2	Aquaporin 2 (collecting duct)
ARRB2	Arrestin, beta 2
CALM2	Calmodulin 2 (phosphorylase kinase, delta)
DLG1	Discs, large homolog 1 (Drosophila)
GCG	Glucagon
GIP	Gastric inhibitory polypeptide
GNA13	Guanine nucleotide binding protein, alpha 13
GNAI1*	Guanine nucleotide binding protein, alpha inhibiting activity polypeptide 1
GNAZ	Guanine nucleotide binding protein, alpha z polypeptide
**GNB4**	**Guanine nucleotide binding protein, beta polypeptide 4**
**GNB5**	**Guanine nucleotide binding protein, beta 5**
**GNG10**	**Guanine nucleotide binding protein, gamma 10**
**GNG11**	**Guanine nucleotide binding protein, gamma 11**
**GNG8**	**Guanine nucleotide binding protein, gamma 8**
GRIK2	Glutamate receptor, ionotropic, kainate 2
IQGAP1	IQ motif containing GTPase activating protein 1
ITPR2	Inositol 1,4,5-trisphosphate receptor, type 2
PIK3CG	Phosphatidylinositol-4,5-bisphosphate 3-kinase, catalytic subunit gamma
PIK3R6	Phosphoinositide-3-kinase, regulatory subunit 6
PLCB1	Phospholipase C, beta 1 (phosphoinositide-specific)
PRKACA	Protein kinase, cAMP-dependent, catalytic, alpha
PRKAR1A*	Protein kinase, cAMP-dependent, regulatory, type I, alpha
PRKAR2A*	Protein kinase, cAMP-dependent, regulatory, type II, alpha
RAP1A	RAP1A, member of RAS oncogene family
RAP1B	RAP1B, member of RAS oncogene family
RHOA*	Ras homolog family member A

* Overlaps with the BioCarta pathways.

**Table 5:** Genes in the leading edge of the incretin-related Reactome pathway. Genes associated with high fatigue are shown in bold.

**Symbol**	**Name**
DPP4	Dipeptidyl-peptidase 4
**FFAR1**	**Free fatty acid receptor 1**
**GATA4**	**GATA binding protein 4**
GCG*	**Glucagon**
GIP*	**Gastric inhibitory polypeptide**
ISL1	ISL LIM homeobox 1
LEP	Leptin
**PCSK1**	**Proprotein convertase subtilisin/kexin type 1**
SEC11C	SEC11 homolog C (*S. cerevisiae*)
SPCS1	Signal peptidase complex subunit 1 homolog (*S. cerevisiae*)
**SPCS3**	**Signal peptidase complex subunit 3 homolog** (*S. cerevisiae*)

* Overlaps with the G-protein signalling leading edge.

### SVM classification of the fatigue groups

**Fig 5:**
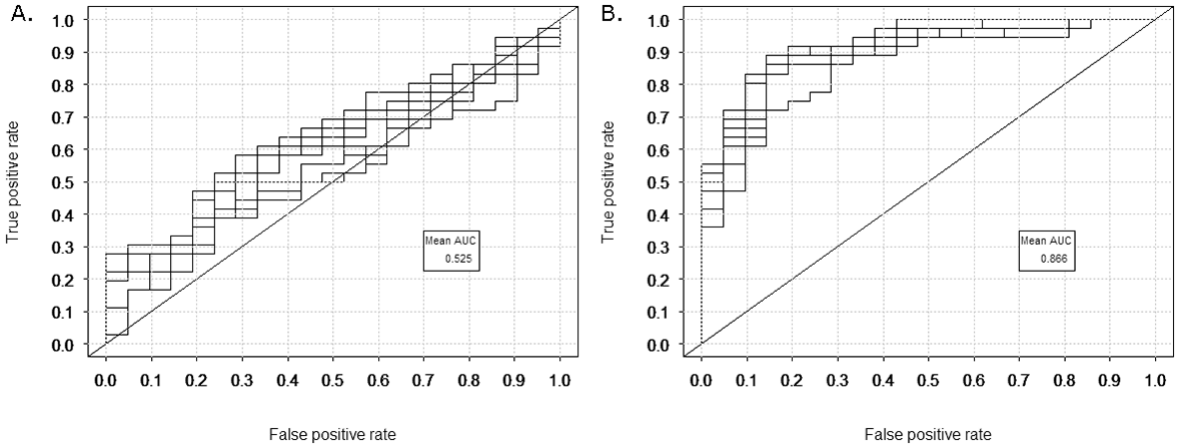
Support vector machine (SVM) classification of fatigue groups. The receiver operator characteristic curves for the SVM output. Ten curves are shown on each plot. The area under the curve (AUC) is calculated as the mean over the ten curves. (A) All 181 enriched pathway genes as input. (B) The 55 leading edge genes as input.

Support vector machines (SVMs) were applied to predict the Fatigue VAS high and low fatigue groups (n = 38 and n = 21, respectively), first using all the genes of the identified pathways as inputs, then using only the 55 leading edge genes. The SVM classifiers were run 10 times, using 10-fold cross-validation over the patient set, producing a mean AUC of 0.525 for all genes and 0.866 for the leading edge genes (Fig 5). The SE(W) values were 0.006 and 0.002, respectively, indicating that the difference in AUC was statistically significant. When the leading edge genes were used as inputs in a classifier of patients and healthy controls the mean AUC was 0.597 with an SE(W) of 0.003. Finally, 50 randomly selected lists of 55 genes were used as inputs into the SVM. AUCs for the random lists had a mean of 0.554 and standard deviation of ±0.080. All of the AUCs for the random gene lists were significantly lower than the AUC for the 55 leading edge genes by SE(W). GSEA using the 55 genes as a bespoke gene set also showed no significant enrichment between patients and healthy controls with an FDR *q*-value of 0.55.

## Discussion

The aim of this study was to analyse the expression of genes between pSS patients discordant for fatigue, in order to identify factors that may be involved in the pathogenesis of fatigue. Extensive subjective and objective clinical data are available for all patients, an ideal basis for the study of fatigue, since it allows accurate assessment of not only the patients’ fatigue status but also their levels of other, possibly confounding, factors. In particular, pain, anxiety and depression have previously been associated with fatigue in pSS [28–30], and may mask fatigue-related associations.

Initially, the microarray data were analysed on a gene by gene basis; no significant changes in expression were detected. Inclusion of other clinical factors into the analysis did not result in the detection of any fatigue-related changes. Moreover, treating fatigue as a continuous, rather than Boolean, variable also resulted in no identification of significant fatigue-related genes. However, although DEGs were not identified between high and low fatigue patients, a large number of DEGs were identified between the patients and controls which were consistent with previously reported DEGs in pSS [31–34]. Additionally, although the IFN signature was not associated with the fatigue groups, it was associated with pSS disease activity, which is also consistent with previous data [21]. Consequently, the lack of significant fatigue-related results is unlikely to be due to data quality or the use of an “atypical” pSS cohort. Indeed, previous studies of chronic fatigue, one using data from monozygotic twins chronic fatigue syndrome (CFS), the other breast cancer patients, also found no significant DEGs [35, 36].

Since fatigue is a complex phenomenon, expression effects may be relatively low in comparison to the noise inherent to high throughput microarray technology, and cannot be detected on an individual gene basis. GSEA is a technique developed to address such situations by detecting subtle changes in pre-determined gene sets [37]. GSEA has the advantage of detecting biological changes that are distributed across a group of genes and, therefore, can identify pathway-level effects. For instance, multiple small changes in a pathway may change its overall metabolic flux leading to a disease state. Moreover, the leading edge of a significant gene set, comprising those genes that account for the enrichment, can include the biologically-relevant subset within a pathway. We applied the GSEA algorithm to the identification of significant enrichment in metabolic pathways: either enrichment in the high or in the low fatigue groups or split distributions between the two groups. Nineteen gene sets from either BioCarta [25] or Reactome [26] were identified as having significant distributions.

The BioCarta pathways enriched in high fatigue, CDC42RAC, MPR and ACTINY, are related pathways associated with actin filaments and migration of cells. The CDC42RAC pathway is involved in several aspect of cell motility including leukocyte movement, fibroblast response and cancer invasiveness [38]. Notably, both CDC42 and RAC1 have been previously associated with CFS in a meta-analysis of multiple data types by Pihur and co-workers [39]. The ACTINY pathway also involves the RAC1 protein to facilitate cell motility via the polymerisation of actin [40]. The third pathway, MPR, involves triggering of the ACTINY pathway by progesterone [41]. These pathways involve N-WASP, the Wiskott-Alrich syndrome-like actin regulating protein, which is known to be highly expressed in neural tissues, associated with T-cell development [42] and involved in actin filament formation in muscle.

At the core of all three BioCarta pathways is the ARP2/3 complex, a major regulator of cell shape and motility via actin cytoskeleton assembly [43]. Seven genes of this complex overlap between the leading edges of the three pathways suggesting that their enrichment in the high fatigue group may indicate a change in ARP2/3 complex activity in these patients. Two of the actin-related genes in the leading edge overlap, ACTR3 and ARPC5, have previously been identified as differentially expressed in CFS by Kerr and colleagues [44], and later confirmed by Zhang and colleagues [45]. APRC5 has also been linked to fatigue in CFS by Frampton and co-workers [46]. In addition, two further genes, which were not in the leading edge overlap, were also identified by previous studies of fatigue in CFS: PIK3RI [44, 45] and PRKAR1A [44, 46, 47].

The 15 Reactome pathways enriched in the high fatigue patient group are all related to guanine nucleotide binding protein (G-protein) signalling and the leading edge overlap comprises five G-protein *β*−/*γ*− subunits, GNB4, GNB5, GNG8, GNG10 and GNG11. G-protein *β* and *γ* subunits are abundant in immune cells [48], and G-protein coupled receptors (GPCRs) have been hypothesised to be involved in fatigue-related disorders [49–51]. In particular, the adrenergic alpha-2A receptor was linked to fatigue in a sub-group of CFS patients by Light and colleagues [50] and several other GPCRs were linked to fatigue by the authors in a later study of prostate cancer and CFS [49]. G-protein *α*-subunits have also been linked to fatigue in CFS [44, 45]. One gene not found in the leading edge overlap, GRIK2, has been linked to CFS [44, 45, 52] and two further genes, PIK3RI and PRKAR1A, are also members of the enriched BioCarta pathways that have previously been linked to fatigue in CFS [44–47]. A fourth gene not found in the overlap, PRKACA, is linked to Cushing’s disease, symptoms of which include severe fatigue [53]. Notably, G-protein signalling pathways have been linked to the cytoskeleton and actin fibres [54, 55], and interact with cytoskeleton regulators [56], consistent with the BioCarta enrichments.

The incretin synthesis, secretion, and inactivation pathway gene set had a non-random distribution of enrichment, indicating that some genes of the pathways are associated with high fatigue, and others with low fatigue. Incretins are produced in the gut, with those entering the bloodstream being rapidly broken down by DPP4 (also known as CD26), a protein found on the surface of T-calls [57]. Inhibitors of DPP4 are used to treat diabetes and can cause fatigue [58], consistent with our observation of an association between DPP4 and low fatigue. Abnormalities in DPP4 levels have also been observed in the autoimmune condition multiple sclerosis, the symptoms of which commonly involve chronic fatigue [59]. Notably, reduced levels of this protein have been identified as a potential biomarker for CFS [60]. However, significant expansion of CD26+ T-cell populations has also been observed in this condition [61]. Another leading edge gene in this pathway, LEP, is involved in the regulation of energy balance, and is linked to several diseases including type 2 diabetes [62]. Fatigue severity has been associated with high circulating levels of this gene’s protein product, leptin, in CFS [63] and in chronic hepatitis [64]. It should also be noted that this pathway is enriched in the pSS group as a whole, indicating this may be a disease-related process rather than specific to fatigue. However, further investigation of the link between this pathway and fatigue is warranted.

The GSEA results were used to select input features for machine learning. Support Vector Machines (SVMs) are machine learning classifiers which aim to separate groups which are non-linearly overlapping using a kernel function to map the data into higher dimensional space [65]. Here, we used a radial kernel SVM to assess the association of the identified pathways with fatigue by comparing the output of classifiers using all enriched pathway genes, with those using only the leading edge genes. ROC curves were used to assess the classifiers’ accuracy, revealing a markedly significant improvement in classifier performance when only the leading edge genes were used as classifier inputs. Further investigation will be required to ascertain the relationship between the enriched pathways and their leading edge genes in order to determine the pathophysiological mechanisms by which these pathways may affect fatigue.

Reliance on patient-reported data is a potential drawback of this study since these patient-reported measures may not be directly comparable due to individual interpretation of the questions. Further, the number of patients per group is relatively small since the fatigue level cutoffs used include only those patients at the extremes of the Fatigue VAS score, which may lower the power of the analysis between high fatigue and low fatigue groups. However, the results of analysing fatigue as a continuous variable, and therefore including the entire patient group, were consistent with the lack of significant DEGs seen between the high and low fatigue extremes. Further, the inclusion of other clinical factors, such as age and depression level, also revealed no significant DEGs. Ultimately, in the absence of an objective measure of fatigue, subjective data must be relied upon. Although the healthy controls were only used in the linear regression model of fatigue as a continuous variable, it should be noted that it is highly unlikely that these individuals would score 0 for fatigue if these data were available. Several of the other clinical factor measurements used in this study are also subjective, and therefore these factors suffer from the same weaknesses as the fatigue score. Consequently, the effects of these possibly confounding factors are unlikely to be eliminated completely. Additionally, the changes observed may be related to differences in white cell count, although the total white cell counts were comparable between the high and low fatigue groups of pSS. Further investigation of these factors is currently underway.

It is likely that fatigue is not a single biological phenomenon in pSS or other fatigue-related disorders. The GSEA results suggest changes in a range of signalling-related processes, potentially indicating multiple pathophysiological mechanisms for the development of fatigue. Stratification of the patients, as is recommended for studies of CFS [66], may therefore aid future studies of fatigue. Future investigation in a larger cohort of pSS patients is in progress and could provide scope for the stratification of patients’ fatigue if required.

Despite these potential limitations, the SVM classifier of fatigue performed well and had significantly improved accuracy over the control classifier. The identified pathways and genes are consistent with several previous studies of fatigue. Furthermore, since the identified genes were neither predictive of pSS nor enriched in the pSS group, they are likely to be related to the fatigue aspect of the disease process. Although overfitting is a possibility, as with all classification techniques, it is unlikely in this case since performance was consistently high on separate testing and training datasets.

The microarray profiling of 133 patients discordant for fatigue has enabled us to identify a 55 genes which are predictive of fatigue in this group. This study provides the first step towards the understanding the underlying mechanisms of fatigue in pSS. Although only a weak signal was observed on a single gene basis, the genes as a group are a strong predictor of fatigue and suggest that a range of signalling changes may be implicated. The relevance of these genes to the pathophysiological mechanisms of fatigue remains to be elucidated. However, the existence and implications of this gene group is of potentially huge importance, and will benefit from further investigation. In particular, this gene list could aid in the future development of objective diagnostics for fatigue-related disorders that are currently non-trivial to diagnose, such as CFS. Whether the gene signature is related to fatigue in general or is specific to pSS-related fatigue should also be investigated in other autoimmune diseases and in CFS itself.

## Materials and Methods

### Patient Recruitment

Contemporaneous patient and healthy control data for this study were obtained from the UK Primary Sjögren’s Syndrome Registry (UKPSSR) [67]. The registry comprises a cohort of clinically well-characterized pSS patients from 30 UK centres with biobanked peripheral blood mononuclear cells, serum, DNA and RNA. Informed written consent was obtained from all patients according to the principles of the Helsinki Declaration. Research Ethical approval for the study was given by the UK National Research Ethics Committee North West—Haydock. All patients fulfilled the American European Consensus Group Criteria (AECG) [20]. Extensive clinical profiles are available for the patients, including demographics, disease activity and damage, past and current treatments, and patient-reported outcome measures. The registry’s healthy controls were recruited at the same time as the patients comprising a group of non-pSS individuals, age-matched to ± 3 years of the patient group. Assessment and sample collection followed a uniform protocol.

A group of 133 pSS patients with variable degrees of fatigue were chosen for the gene expression study. Additionally, 29 healthy individuals also selected from the cohort as a control ensuring no history of fatigue, dry eyes/mouth or autoimmune disease. Peripheral blood samples were collected and kept in PAXgene blood RNA tubes (Becton, Dickinson and Company, Oxford), which contain blood cell-lysing and RNA-stabilizing reagents. Samples were stored at −80°C prior to RNA extraction.

### Laboratory Techniques

RNA was extracted from peripheral whole blood samples using the PAXgene Blood miRNA kit (PreAnalytix GmbH, Switzerland). The extractions were performed according to the manufacturer’s protocol. The RNeasy MiniElute kit (QIAGEN, Manchester) was used to obtain the required concentration and volume for the Globin mRNA reduction procedure.

Globin mRNA was removed from the RNA samples using the Human GLOBINclear kit (Ambion Inc., Texas, USA). The purity and the concentration of the globin-cleared samples were assessed using the Nano-drop ND-1000 spectrophotometer (Willmington, USA). The samples were stored at −20°C, according to the GLOBINclear manufacturer’s protocol [68]. The quality of all samples was analysed with the Agilent 2100 Bioanalyzer using the Agilent RNA Nano kit (Agilent, Santa Clara, USA). Samples with a RNA integrity number (RIN) of above seven were used for whole genome microarray using the Illumina HumanHT-12 v4 BeadChip. Both techniques were performed at Cambridge Genomic Services (Cambridge, UK).

### Fatigue and Other Clinical Factors

Fatigue was defined using the patient-reported abnormal fatigue as scored on a visual analogue scale of 0–100 [69]. Patients were considered “high fatigue” with a score >75 and “low fatigue” <25.

Several other factors were included in the linear fits:

- *Depression* and *anxiety*: measured using the Hospital Anxiety and Depression (HAD) scale [70]
- *Pain* and *dryness*: measured using the ESSPRI pain and dryness sub-domains [71]
- *Age* at cohort recruitment (the date of blood sample collection)
- *Disease activity* measured using the EULAR Sjögren’s Syndrome Disease Activity Index (ESSDAI) [72]
- *Disease damage* measured using the Sjögren’s Syndrome Disease Damage Index (SSDDI) [73]

### Gene Expression Analysis

Gene expression data were prepared for analysis using the microarray packages provided by BioConductor [74] as described by Cockell and colleagues [75]. Data were transformed to stabilise the variance across probes before robust spline normalisation using the lumi package [76]. The arrayQualityMetrics package was used to detect outliers [77]. The lumi command detectionCall was used to filter out probes with a detection *p*-value less than 0.01. This filtering step was not included prior to gene set enrichment analysis (GSEA) since the algorithm requires unfiltered data [37]. Batch effects were removed using the combat package [78]. Gene annotations were retrieved from the lumiHumanAll.db package [79].

**Fig 6:**
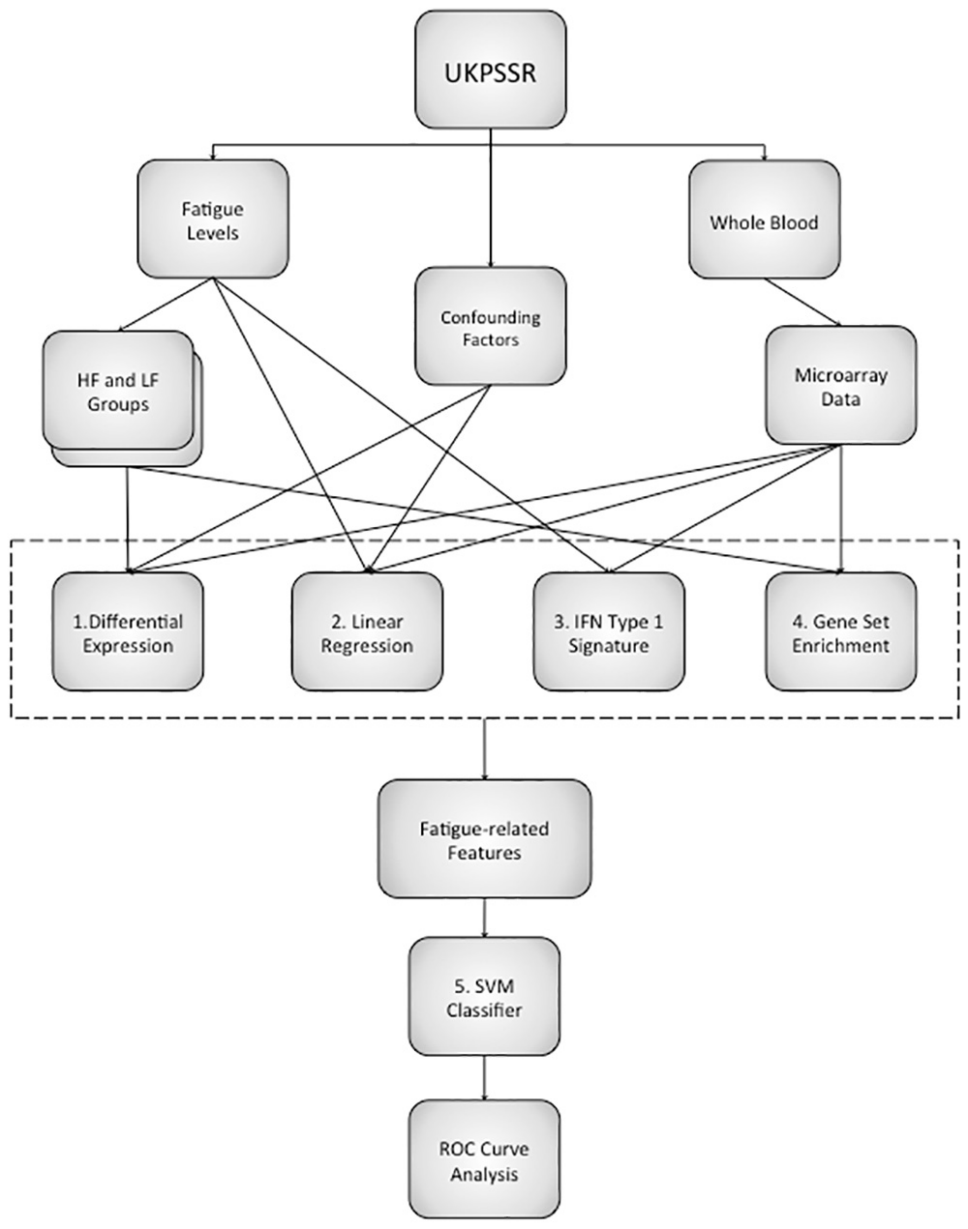
A workflow of the gene expression analysis. The gene expression data were analysed to produce a list of fatigue-related features which were used as inputs for a support vector machine classifier of fatigue. 1. Differentially expressed genes were identified between fatigue groups. 2. Linear regression was used to analyse fatigue as a continuous variable. 3. The interferon type I signature was calculated for all the patients and compared to fatigue levels. 4. Gene set enrichment analysis was carried out using the high and low fatigue groups. 5. A support vector machine classifier was created using fatigue-related features as inputs and its performance assessed using receiver-operator characteristic (ROC) curves.

The expression data were then analysed using several parallel approaches (Fig 6):

1. Differentially expressed genes between “high fatigue” and “low fatigue” pSS patients were identified using the limma package [80] at a fold-change cutoff of 1.2 and a *p*-value cutoff of 0.05 after adjustment using the Benjamini-Hochberg false discovery rate [81]. Other clinical factors were corrected for by inclusion in the linear fits.
2. The Fatigue VAS scores were analysed as a continuous variable by fitting a linear regression model to the expression data including both the pSS and healthy control groups. Since fatigue data were not available for the controls, their individual scores were considered 0. Other clinical factors were corrected for by inclusion in the regression models. The *p*-values were adjusted using the Benjamini-Hochberg false discovery rate [81] and a *p*-value significance cutoff of 0.05 was applied.
3. The IFN type I signature was calculated for all the patients based on the five INF induced genes identified by Brkic and colleagues [21]. Scores were calculated for each patient as the number of healthy control standard deviations above the healthy control mean, summed over all five genes, as described by Kirou and co-workers [82]. Patients with a score exceeding 10 were considered to be IFN-positive [21].
4. GSEA and leading edge analysis were carried out using the GSEA software package [37, 83]. Gene sets were taken from version 4 of the Molecular Signature Database (MSigDB) [24]. All 1320 canonical pathway gene sets (collection C2:CP) were tested. Additionally, the fatigue-related features identified (point 5) were analysed as a bespoke input gene set. Gene sets were considered significant at an FDR cut-off of 25%. Real gene ordering was used to detect enrichments in the low and high groups, while absolute gene ordering was used to detect other non-random distributions.
5. Machine learning was carried out on the high and low fatigue groups using radial kernel support vector machines (SVMs) [84] run in the e1071 package [85]. Hyperparameter inputs were selected and inputs pre-processed using the carat package [86] and 10-fold cross-validation was applied. The performance of the classifiers was evaluated using the area under curve (AUC) of receiver operator characteristic (ROC) curves [87]. The error of the AUC was calculated using the standard error of the Wilcoxon statistic SE(W) [87, 88] using Eq (1), where *θ* is the AUC, *C*_*p*_ is the number of positive examples, *C*_*n*_ is the number of negative examples, and *Q*_1_ and *Q*_2_ are the probabilities of incorrect group assignment as defined by Eqs (2) and (3), respectively.
  $$SE\left(W\right)=\sqrt{\frac{\theta \left(1-\theta \right)+\left(C_{p}-1\right)\left(Q_{1}-\theta^{2}\right)+\left(C_{n}-1\right)\left(Q_{2}-\theta^{2}\right)}{C_{p}C_{n}}}$$ (1)
  $$Q_{1}=\frac{\theta }{2-\theta }$$ (2)
  $$Q_{2}=\frac{2\theta^{2}}{1+\theta }$$ (3)

## Supporting Information

S1 Table Correlations between fatigue and clinical factors. The correlations between the three fatigue scores and the other clinical factors included in the analyses.
(DOCX)

S2 Table Fatigue as a continuous variable. The top 10 genes from the linear fits of the three fatigue scores. In all three cases no genes were statistically significant after *p*-value adjustment.
(DOCX)

S3 Table Correction for clinical factors. The top five genes for the linear fits of the three fatigue scores corrected for the other clinical factors. Factors were included in the regression fits individually and in combination. No significantly differentially expressed genes were found. Disease activity was measured using the EULAR Sjögren’s Syndrome Disease Activity Index. Disease damage was measured using the Sjögren’s Syndrome Disease Damage Index. Dryness and pain were measured using the EULAR Sjögren’s Syndrome Patient Reported Index dryness and pain sub-domains, respectively. Anxiety and depression were measured using the Hospital Anxiety and Depression scale.
(DOCX)

S4 Table Enriched pathways in pSS. Gene sets were considered to be enriched at an FDR cut-off of 25%.
(DOCX)

S1 Fig Outlier Detection. Bar charts of the three outlier detection methods. In each case the bars are shown in the original order of the arrays. Two arrays, numbers 61 and 121, were identified as outliers (red crosses). A) Hoeffding’s statistic *D*_*a*_. A threshold of 0.15 was used, which is indicated by the vertical line. No arrays exceeded the outlier threshold. B) The sum of distances to other arrays *S*_*a*_. Based on the distribution of the values across all arrays, a threshold of 34.8 was determined, which is indicated by the vertical line. Two arrays significantly exceeded the threshold and were considered outliers. C) The Kolmogorov-Smirnov statistic *K*_*a*_. Based on the distribution of the values across all arrays, a threshold of 0.0558 was determined, which is indicated by the vertical line. One array significantly exceeded this threshold and was considered an outlier.
(PNG)

S2 Fig Batch correction. Principle component plots of the data pre- (A) and post-batch correction (B). Points are coloured and shaped by experimental batch.
(PNG)

S3 Fig Differential gene expression analysis. Volcano plots for fatigue groups using PROFAD and ESSPRI fatigue scores. The ranges of these scores are 0–7 for PROFAD and 0–10 for ESSPRI, respectively. No significantly differentially expressed genes were identified in either case. A. PROFAD, high fatigue >5 (n = 32) and low fatigue ≤2 (n = 32). B. ESSPRI, high fatigue >7 (n = 36) and low fatigue ≤3 (n = 34).
(PNG)

S4 Fig PROFAD correction for clinical factors. Volcano plots for the PROFAD fatigue groups corrected for clinical factors. High fatigue >5 (n = 32) and low fatigue ≤2 (n = 32). A. Age at UKPSSR cohort recruitment. B. Disease activity measured using the EULAR Sjögren’s Syndrome Disease Activity Index. C. Disease damage measured using the Sjögren’s Syndrome Disease Damage Index. D. The EULAR Sjögren’s Syndrome Patient Reported Index dryness sub-domain. E. The EULAR Sjögren’s Syndrome Patient Reported Index pain sub-domain. F. Anxiety measured using the Hospital Anxiety and Depression scale. G. Depression measured using the Hospital Anxiety and Depression scale. H. Pain and depression (E & G). I. Pain, depression, dryness and anxiety (D-G). J. All seven factors (A-G). No significantly differentially expressed genes were identified following any correction.
(PNG)

S5 Fig ESSPRI correction for clinical factors. Volcano plots for the ESSPRI physical fatigue groups corrected for clinical factors. High fatigue >7 (n = 36) and low fatigue ≤3 (n = 34). A. Age at UKPSSR cohort recruitment. B. Disease activity measured using the EULAR Sjögren’s Syndrome Disease Activity Index. C. Disease damage measured using the Sjögren’s Syndrome Disease Damage Index. D. The EULAR Sjögren’s Syndrome Patient Reported Index dryness sub-domain. E. The EULAR Sjögren’s Syndrome Patient Reported Index pain sub-domain. F. Anxiety measured using the Hospital Anxiety and Depression scale. G. Depression measured using the Hospital Anxiety and Depression scale. H. Pain and depression (E & G). I. Pain, depression, dryness and anxiety (D-G). J. All seven factors (A-G). No significantly differentially expressed genes were identified following any correction.
(PNG)

S6 Fig Interferon type I signature. The clinical scores in the IFN type I positive and negative groups. ESSDAI scores were significantly higher in the IFN positive group. However, there was no significant relationship between IFN signature and ESSPRI, SSDDI or the three fatigue scores.
(PNG)
