# Supplementary material for: A Transcriptional Signature of Fatigue Derived from Patients with Primary Sjögren’s Syndrome
Source: PLoS One. 2015 Dec 22;10(12):e0143970. doi: 10.1371/journal.pone.0143970 (PMC4687914; doi:10.1371/journal.pone.0143970)
Supplement: S1 Table — The correlations between the three fatigue scores and the other clinical factors included in the analyses. (DOCX) [file pone.0143970.s001.docx]

**Table S1 Correlations between fatigue and clinical factors.** The correlations between the three fatigue scores and the other clinical factors included in the analyses.

| **Factor** | **Fatigue VAS** | **PROFAD Physical Fatigue** | **ESSPRI Fatigue** |
| --- | --- | --- | --- |
| ESSPRI Pain | 0.616 | 0.693 | 0.644 |
| ESSPRI Dryness | 0.47 | 0.518 | 0.485 |
| HAD Depression | 0.642 | 0.672 | 0.613 |
| HAD Anxiety | 0.451 | 0.442 | 0.397 |
| Age at Recruitment | 0.105 | 0.015 | 0.087 |
| ESSDAI | 0.167 | 0.18 | 0.129 |
| SSDDI | -0.074 | -0.079 | -0.076 |

ESSDAI = EULAR Sjögren's Syndrome Disease Activity Index, SSDDI = Sjögren's Syndrome Disease Damage Index, ESSPRI = EULAR Sjögren's Syndrome Patient Reported Index, HAD = Hospital Anxiety and Depression, PROFAD = Profile of Fatigue and Discomfort.
