# Supplementary material for: A Transcriptional Signature of Fatigue Derived from Patients with Primary Sjögren’s Syndrome
Source: PLoS One. 2015 Dec 22;10(12):e0143970. doi: 10.1371/journal.pone.0143970 (PMC4687914; doi:10.1371/journal.pone.0143970)
Supplement: S2 Table — The top 10 genes from the linear fits of the three fatigue scores. In all three cases no genes were statistically significant after p-value adjustment. (DOCX) [file pone.0143970.s002.docx]

**Table S6. Fatigue as a continuous variable.** The top 10 genes from the linear fits of the three fatigue scores. In all three cases no genes were statistically significant after *p*-value adjustment.

| **Fatigue VAS** | | | **PROFAD Physical Fatigue** | | | **ESSPRI Fatigue** | | |
| --- | --- | --- | --- | --- | --- | --- | --- | --- |
| **Symbol** | ***P-*value** | **Adjusted *P*-value** | **Symbol** | ***P-*value** | **Adjusted *P*-value** | **Symbol** | ***P-*value** | **Adjusted *P*-value** |
| PAXBP1 | 9.08E-05 | 1.00E+00 | PAXBP1 | 7.96E-05 | 6.10E-01 | LRRC8E | 1.47E-04 | 9.98E-01 |
| ERAP1 | 2.56E-04 | 1.00E+00 | ZNF714 | 1.02E-04 | 6.10E-01 | ZNF714 | 4.93E-04 | 9.98E-01 |
| CST4 | 6.43E-04 | 1.00E+00 | NFIA | 1.44E-04 | 6.10E-01 | AMDHD1 | 6.07E-04 | 9.98E-01 |
| AVL9 | 8.04E-04 | 1.00E+00 | SMUG1 | 1.45E-04 | 6.10E-01 | CTSG | 8.51E-04 | 9.98E-01 |
| ASB8 | 9.17E-04 | 1.00E+00 | MEF2C | 1.93E-04 | 6.10E-01 | RNASE3 | 1.09E-03 | 9.98E-01 |
| ZNF10 | 9.31E-04 | 1.00E+00 | RNF170 | 2.66E-04 | 7.13E-01 | PAXBP1 | 1.22E-03 | 9.98E-01 |
| MSANTD3 | 9.36E-04 | 1.00E+00 | ELOVL6 | 2.80E-04 | 7.13E-01 | SMUG1 | 1.23E-03 | 9.98E-01 |
| C4orf3 | 9.54E-04 | 1.00E+00 | SDC4 | 3.00E-04 | 7.13E-01 | NAPG | 1.53E-03 | 9.98E-01 |
| LRRC8E | 9.55E-04 | 1.00E+00 | COQ4 | 4.39E-04 | 8.01E-01 | SIGLEC8 | 1.56E-03 | 9.98E-01 |
| BAMBI | 1.03E-03 | 1.00E+00 | B4GALNT4 | 4.65E-04 | 8.01E-01 | BAIAP2 | 1.68E-03 | 9.98E-01 |
