# Supplementary material for: A Transcriptional Signature of Fatigue Derived from Patients with Primary Sjögren’s Syndrome
Source: PLoS One. 2015 Dec 22;10(12):e0143970. doi: 10.1371/journal.pone.0143970 (PMC4687914; doi:10.1371/journal.pone.0143970)
Supplement: S3 Table — The top five genes for the linear fits of the three fatigue scores corrected for the other clinical factors. Factors were included in the regression fits individually and in combination. No significantly differentially expressed genes were found. Disease activity was measured using the EULAR Sjögren’s Syndrome Disease Activity Index. Disease damage was measured using the Sjögren’s Syndrome Disease Damage Index. Dryness and pain were measured using the EULAR Sjögren’s Syndrome Patient Reported Index dryness and pain sub-domains, respectively. Anxiety and depression were measured using the Hospital Anxiety and Depression scale. (DOCX) [file pone.0143970.s003.docx]

**Table S7 Correction for confounders.** The top five genes for the linear fits of the three fatigue scores corrected for the confounding factors. Confounders were included in the regression fits individually and in combination. No significantly differentially expressed genes were found. Disease activity is measured using the EULAR Sjögren's Syndrome Disease Activity Index. Disease damage is measured using the Sjögren's Syndrome Disease Damage Index. Dryness and pain are measured using the EULAR Sjögren's Syndrome Patient Reported Index dryness and pain subdomains. Anxiety and depression are measured using the Hospital Anxiety and Depression scale.

|  | **Fatigue VAS** | | | **PROFAD Physical Fatigue** | | | **ESSPRI Fatigue** | | |
| --- | --- | --- | --- | --- | --- | --- | --- | --- | --- |
|  | **Symbol** | ***P*-value** | **Adjusted *P*-value** | **Symbol** | ***P*-value** | **Adjusted *P*-value** | **Symbol** | ***P*-value** | **Adjusted *P*-value** |
| **Age at Recruitment** | PAXBP1 | 8.64E-05 | 1.00E+00 | PAXBP1 | 8.34E-05 | 5.73E-01 | LRRC8E | 1.95E-04 | 9.97E-01 |
|  | ERAP1 | 2.29E-04 | 1.00E+00 | ZNF714 | 1.03E-04 | 5.73E-01 | CTSG | 2.62E-04 | 9.97E-01 |
|  | ZNF10 | 6.61E-04 | 1.00E+00 | NFIA | 1.42E-04 | 5.73E-01 | ZNF714 | 3.86E-04 | 9.97E-01 |
|  | C4orf3 | 6.78E-04 | 1.00E+00 | SMUG1 | 1.48E-04 | 5.73E-01 | RNASE3 | 4.22E-04 | 9.97E-01 |
|  | AVL9 | 6.79E-04 | 1.00E+00 | MEF2C | 1.57E-04 | 5.73E-01 | SDC4 | 5.89E-04 | 9.97E-01 |
| **Disease Activity** | PAXBP1 | 5.27E-05 | 1.00E+00 | PAXBP1 | 4.74E-05 | 5.53E-01 | LRRC8E | 6.30E-05 | 1.00E+00 |
|  | ERAP1 | 1.69E-04 | 1.00E+00 | MEF2C | 1.68E-04 | 6.93E-01 | BAIAP2 | 5.39E-04 | 1.00E+00 |
|  | LRRC8E | 3.72E-04 | 1.00E+00 | ZNF714 | 1.80E-04 | 6.93E-01 | ZNF714 | 7.27E-04 | 1.00E+00 |
|  | ASB8 | 6.02E-04 | 1.00E+00 | NFIA | 1.95E-04 | 6.93E-01 | PAXBP1 | 9.04E-04 | 1.00E+00 |
|  | OXT | 7.01E-04 | 1.00E+00 | SEMA5B | 2.19E-04 | 6.94E-01 | AMDHD1 | 9.14E-04 | 1.00E+00 |
| **Disease Damage** | PAXBP1 | 1.08E-04 | 9.99E-01 | SMUG1 | 8.63E-05 | 5.03E-01 | LRRC8E | 1.89E-04 | 9.98E-01 |
|  | ERAP1 | 3.71E-04 | 9.99E-01 | ZNF714 | 8.82E-05 | 5.03E-01 | RNASE3 | 3.77E-04 | 9.98E-01 |
|  | CST4 | 4.48E-04 | 9.99E-01 | PAXBP1 | 9.34E-05 | 5.03E-01 | CTSG | 3.77E-04 | 9.98E-01 |
|  | AK2 | 5.46E-04 | 9.99E-01 | MEF2C | 1.62E-04 | 6.59E-01 | ZNF714 | 4.50E-04 | 9.98E-01 |
|  | BICD2 | 6.03E-04 | 9.99E-01 | NFIA | 1.86E-04 | 6.63E-01 | AMDHD1 | 6.45E-04 | 9.98E-01 |
| **Dryness** | AVL9 | 3.53E-05 | 1.00E+00 | ZNF714 | 9.30E-06 | 2.65E-01 | ZNF714 | 9.41E-05 | 1.00E+00 |
|  | ERAP1 | 8.47E-05 | 1.00E+00 | SEMA5B | 1.22E-04 | 9.25E-01 | PAX7 | 1.35E-04 | 1.00E+00 |
|  | PAXBP1 | 2.17E-04 | 1.00E+00 | OR4C13 | 1.31E-04 | 9.25E-01 | LRRC8E | 2.14E-04 | 1.00E+00 |
|  | ASB8 | 2.82E-04 | 1.00E+00 | PAXBP1 | 1.62E-04 | 9.25E-01 | PLAC1 | 5.47E-04 | 1.00E+00 |
|  | ZNF714 | 2.88E-04 | 1.00E+00 | ASB8 | 3.63E-04 | 1.00E+00 | AVL9 | 9.45E-04 | 1.00E+00 |
| **Pain** | NFKBIB | 3.70E-04 | 1.00E+00 | COQ4 | 8.71E-05 | 1.00E+00 | HMBOX1 | 1.40E-04 | 1.00E+00 |
|  | ZNF705A | 4.36E-04 | 1.00E+00 | KIR2DS1 | 2.08E-04 | 1.00E+00 | NT5DC3 | 7.21E-04 | 1.00E+00 |
|  | LMOD2 | 4.56E-04 | 1.00E+00 | HMBOX1 | 3.37E-04 | 1.00E+00 | PER2 | 9.48E-04 | 1.00E+00 |
|  | CST4 | 5.11E-04 | 1.00E+00 | TCF7L2 | 3.50E-04 | 1.00E+00 | KIR3DS1 | 1.07E-03 | 1.00E+00 |
|  | HMBOX1 | 6.34E-04 | 1.00E+00 | IFFO1 | 6.43E-04 | 1.00E+00 | RNASE1 | 1.08E-03 | 1.00E+00 |
| **Anxiety** | BAMBI | 3.05E-05 | 8.69E-01 | SMUG1 | 7.10E-05 | 4.11E-01 | AMDHD1 | 4.89E-04 | 9.94E-01 |
|  | AK2 | 2.53E-04 | 1.00E+00 | RNF170 | 7.20E-05 | 4.11E-01 | CTSG | 5.04E-04 | 9.94E-01 |
|  | RNF170 | 4.92E-04 | 1.00E+00 | B4GALNT4 | 8.90E-05 | 4.23E-01 | NUDT3 | 5.50E-04 | 9.94E-01 |
|  | AVL9 | 8.51E-04 | 1.00E+00 | MEF2C | 1.09E-04 | 4.45E-01 | NAV2 | 6.05E-04 | 9.94E-01 |
|  | SMUG1 | 1.04E-03 | 1.00E+00 | ZNF714 | 1.56E-04 | 5.39E-01 | LRRC8E | 6.94E-04 | 9.94E-01 |
| **Depression** | KIF25 | 1.14E-04 | 8.42E-01 | EEF1DP3 | 6.93E-05 | 1.00E+00 | KIR3DL3 | 3.00E-04 | 1.00E+00 |
|  | DPP8 | 3.06E-04 | 8.42E-01 | ZNF630 | 1.32E-04 | 1.00E+00 | LOC646670 | 3.65E-04 | 1.00E+00 |
|  | ARNT | 5.08E-04 | 8.42E-01 | BMF | 1.58E-04 | 1.00E+00 | SIGLEC8 | 3.77E-04 | 1.00E+00 |
|  | MSANTD3 | 5.44E-04 | 8.42E-01 | VPS25 | 2.46E-04 | 1.00E+00 | BMF | 5.06E-04 | 1.00E+00 |
|  | AVL9 | 5.53E-04 | 8.42E-01 | MSANTD3 | 2.75E-04 | 1.00E+00 | UNC13A | 6.84E-04 | 1.00E+00 |
| **Pain & Depression** | KIF25 | 8.46E-05 | 6.86E-01 | SLC2A9 | 1.01E-04 | 1.00E+00 | LOC728503 | 1.30E-04 | 9.99E-01 |
|  | JAZF1 | 1.53E-04 | 6.86E-01 | GALR2 | 1.68E-04 | 1.00E+00 | KIR3DL3 | 1.71E-04 | 9.99E-01 |
|  | SULT1A3 | 1.61E-04 | 6.86E-01 | KIR3DL3 | 2.18E-04 | 1.00E+00 | SLC2A9 | 2.07E-04 | 9.99E-01 |
|  | LRRCC1 | 2.08E-04 | 6.86E-01 | MTRF1L | 2.28E-04 | 1.00E+00 | KIR3DS1 | 4.16E-04 | 9.99E-01 |
|  | ZNF705A | 2.14E-04 | 6.86E-01 | FAM156A | 2.94E-04 | 1.00E+00 | HS6ST2-AS1 | 5.07E-04 | 9.99E-01 |
| **Pain, Depression, Anxiety & Dryness** | JAZF1 | 3.30E-05 | 5.56E-01 | KIR3DL3 | 2.59E-04 | 1.00E+00 | YOD1 | 1.03E-04 | 7.10E-01 |
|  | PTPN2 | 3.96E-05 | 5.56E-01 | SLC2A9 | 2.75E-04 | 1.00E+00 | SLC14A1 | 1.11E-04 | 7.10E-01 |
|  | KIF25 | 6.09E-05 | 5.56E-01 | GALR2 | 2.82E-04 | 1.00E+00 | LOC728503 | 1.34E-04 | 7.10E-01 |
|  | YOD1 | 8.17E-05 | 5.56E-01 | MTRF1L | 2.85E-04 | 1.00E+00 | RAB2B | 1.35E-04 | 7.10E-01 |
|  | AVL9 | 1.53E-04 | 5.56E-01 | FAM156A | 3.69E-04 | 1.00E+00 | RIOK3 | 1.70E-04 | 7.10E-01 |
| **All 7 confoundersd** | JAZF1 | 4.09E-05 | 3.30E-01 | SLC2A9 | 1.81E-04 | 9.94E-01 | YOD1 | 6.38E-05 | 5.93E-01 |
|  | PTPN2 | 4.09E-05 | 3.30E-01 | ACOT11 | 2.62E-04 | 9.94E-01 | NAGA | 6.71E-05 | 5.93E-01 |
|  | YOD1 | 4.71E-05 | 3.30E-01 | CPT2 | 2.70E-04 | 9.94E-01 | RIOK3 | 1.18E-04 | 5.93E-01 |
|  | MKL1 | 5.12E-05 | 3.30E-01 | SULT1A3 | 3.16E-04 | 9.94E-01 | SLC2A9 | 1.37E-04 | 5.93E-01 |
|  | KIF25 | 5.97E-05 | 3.30E-01 | MTRF1L | 3.28E-04 | 9.94E-01 | RAB2B | 1.67E-04 | 5.93E-01 |
