# Supplementary material for: A Transcriptional Signature of Fatigue Derived from Patients with Primary Sjögren’s Syndrome
Source: PLoS One. 2015 Dec 22;10(12):e0143970. doi: 10.1371/journal.pone.0143970 (PMC4687914; doi:10.1371/journal.pone.0143970)
Supplement: S4 Table — Gene sets were considered to be enriched at an FDR cut-off of 25%. (DOCX) [file pone.0143970.s004.docx]

**Table S9 Enriched pathways in primary Sjögren’s Syndrome.** Gene sets were considered to be enriched at an FDR cut-off of 25 %.

| **Name** | **Size** | **ES** | **NES** | **NOM *p*-value** | **FDR *q*-value** |
| --- | --- | --- | --- | --- | --- |
| **Biocarta** |  |  |  |  |  |
| Intrinsic pathway | 23 | 0.6710 | 1.8271 | 0.0000 | 0.0153 |
| COMP pathway | 18 | 0.7155 | 1.7036 | 0.0120 | 0.0532 |
| LAIR pathway | 17 | 0.5847 | 1.4443 | 0.0346 | 0.2395 |
| **KEGG** |  |  |  |  |  |
| Cytosolic DNA-sensing pathway | 56 | 0.7336 | 2.0374 | 0.0000 | 0.0000 |
| RIG-I-like receptor signaling pathway | 71 | 0.7229 | 1.9605 | 0.0000 | 0.0018 |
| NOD-like receptor signaling pathway | 62 | 0.6062 | 1.6225 | 0.0080 | 0.1044 |
| Complement and coagulation cascades | 68 | 0.5566 | 1.6070 | 0.0121 | 0.1046 |
| ABC transporters | 44 | 0.4965 | 1.6300 | 0.0000 | 0.1162 |
| Systemic lupus erythematosus | 133 | 0.5726 | 1.6448 | 0.0059 | 0.1251 |
| Proteasome | 44 | 0.6830 | 1.6512 | 0.0060 | 0.1582 |
| **Reactome** |  |  |  |  |  |
| TRAF6 mediated IRF7 activation | 30 | 0.8163 | 1.9534 | 0.0000 | 0.0195 |
| Autodegradation of Cdh1 by Cdh1:APC/C | 56 | 0.6677 | 1.7827 | 0.0020 | 0.0235 |
| Antigen processing: cross presentation | 72 | 0.7081 | 1.8674 | 0.0000 | 0.0247 |
| Innate immune system | 268 | 0.5944 | 1.7830 | 0.0000 | 0.0252 |
| Antiviral mechanism by IFN-stimulated genes | 65 | 0.8041 | 1.7884 | 0.0000 | 0.0255 |
| Interferon signaling | 152 | 0.8436 | 1.7695 | 0.0000 | 0.0258 |
| Regulation of mitotic cell cycle | 77 | 0.6554 | 1.7982 | 0.0000 | 0.0267 |
| ORC1 removal from chromatin | 65 | 0.6262 | 1.7623 | 0.0039 | 0.0268 |
| Regulation of IFNA signaling | 24 | 0.8078 | 1.8788 | 0.0000 | 0.0270 |
| SCF(Skp2) mediated degradation of p27/p21 | 53 | 0.6754 | 1.7902 | 0.0020 | 0.0271 |
| Cyclin E associated events during G1/S transition | 62 | 0.6531 | 1.8005 | 0.0020 | 0.0287 |
| Cross presentation of soluble exogenous antigens endosomes | 47 | 0.7251 | 1.7474 | 0.0020 | 0.0304 |
| TRAF6 mediated NF-Kb activation | 21 | 0.7683 | 1.7323 | 0.0000 | 0.0306 |
| Synthesis secretion and deacylation of ghrelin | 16 | 0.7431 | 1.8031 | 0.0020 | 0.0308 |
| SCF-beta-TrCP mediated degradation of Emi1 | 49 | 0.6723 | 1.7330 | 0.0019 | 0.0316 |
| Negative regulators of RIG-I/MDA5 signaling | 30 | 0.8202 | 1.7339 | 0.0000 | 0.0330 |
| CDT1 association with the CDC6:ORC:origin complex | 54 | 0.6434 | 1.7362 | 0.0019 | 0.0337 |
| APC/C:Cdc20 mediated degradation of mitotic proteins | 65 | 0.6686 | 1.8049 | 0.0000 | 0.0338 |
| G1/S transition | 106 | 0.5859 | 1.7178 | 0.0080 | 0.0342 |
| ER-phagosome pathway | 58 | 0.6867 | 1.8807 | 0.0020 | 0.0349 |
| Cytokine signaling in immune system | 262 | 0.7446 | 1.8116 | 0.0000 | 0.0356 |
| Assembly of the pre-replicative complex | 63 | 0.6126 | 1.7074 | 0.0020 | 0.0372 |
| RIG-I/MDA5 mediated induction of IFN-alpha/beta pathways | 72 | 0.7516 | 1.9658 | 0.0000 | 0.0373 |
| Cell cycle checkpoints | 111 | 0.5671 | 1.6857 | 0.0040 | 0.0389 |
| p53-Independent G1/S DNA damage checkpoint | 48 | 0.6467 | 1.6998 | 0.0020 | 0.0389 |
| G1/S specific transcription | 17 | 0.7331 | 1.6872 | 0.0079 | 0.0396 |
| Destabilization of mRNA by AUF1 (hnRNP D0) | 50 | 0.6229 | 1.6938 | 0.0040 | 0.0397 |
| CDK-mediated phosphorylation and removal of Cdc6 | 46 | 0.6497 | 1.6894 | 0.0039 | 0.0401 |
| APC/C:Cdh1 mediated degradation of Cdc20 and other APC/C:Cdh1 targeted proteins in late mitosis/early G1 | 64 | 0.6792 | 1.8117 | 0.0020 | 0.0412 |
| Synthesis of DNA | 90 | 0.5829 | 1.6716 | 0.0080 | 0.0430 |
| APC/C:Cdc20 mediated degradation of Cyclin B | 19 | 0.6767 | 1.6723 | 0.0098 | 0.0438 |
| M/G1 transition | 78 | 0.5813 | 1.6569 | 0.0059 | 0.0450 |
| Antigen presentation: folding, assembly and peptide loading of class I MHC | 20 | 0.7148 | 1.6643 | 0.0102 | 0.0453 |
| Vif-mediated degradation of APOBEC3G | 49 | 0.6857 | 1.6572 | 0.0020 | 0.0462 |
| S phase | 106 | 0.5628 | 1.6432 | 0.0121 | 0.0471 |
| Cyclin A/B1 associated events during G2/M transition | 15 | 0.6873 | 1.6461 | 0.0174 | 0.0472 |
| p53-dependent G1 DNA damage response | 53 | 0.6097 | 1.6575 | 0.0020 | 0.0475 |
| Class I MHC mediated antigen processing & presentation | 236 | 0.4985 | 1.6396 | 0.0000 | 0.0478 |
| Activation of NF-kappaB in B Cells | 61 | 0.5726 | 1.6468 | 0.0101 | 0.0483 |
| Autodegradation of the E3 ubiquitin ligase COP1 | 47 | 0.6261 | 1.6286 | 0.0080 | 0.0523 |
| Interferon gamma signaling | 58 | 0.8375 | 1.6164 | 0.0000 | 0.0577 |
| Nucleotide binding domain leucine rich repeat containing receptor (NLR) signaling pathways | 45 | 0.6127 | 1.6120 | 0.0041 | 0.0589 |
| Conversion from APC/C:Cdc20 to APC/C:Cdh1 in late anaphase | 16 | 0.6961 | 1.6033 | 0.0040 | 0.0605 |
| Mitotic G1-G1/S phases | 130 | 0.5172 | 1.6038 | 0.0080 | 0.0615 |
| Synthesis secretion and inactivation of glucagon-like peptide-1 (GLP-1) | 18 | 0.5875 | 1.6051 | 0.0297 | 0.0619 |
| Regulation of apoptosis | 56 | 0.5757 | 1.5990 | 0.0099 | 0.0621 |
| Cell cycle | 397 | 0.4456 | 1.5939 | 0.0020 | 0.0621 |
| NOD1/2 | 30 | 0.6223 | 1.5958 | 0.0144 | 0.0626 |
| DNA replication | 186 | 0.5047 | 1.5853 | 0.0120 | 0.0650 |
| Regulation of ornithine decarboxylase (ODC) | 48 | 0.6099 | 1.5868 | 0.0061 | 0.0652 |
| Deposition of new CENPA-containing nucleosomes at the centromere | 62 | 0.5571 | 1.5695 | 0.0472 | 0.0705 |
| Inflammasomes | 16 | 0.7257 | 1.5702 | 0.0158 | 0.0713 |
| Incretin synthesis, secretion, and inactivation | 21 | 0.5428 | 1.5738 | 0.0422 | 0.0715 |
| Meiosis | 110 | 0.4782 | 1.5651 | 0.0421 | 0.0722 |
| Signaling by Wnt | 62 | 0.5545 | 1.5705 | 0.0178 | 0.0724 |
| RIP-mediated NFkB activation via DAI | 18 | 0.6432 | 1.5625 | 0.0243 | 0.0727 |
| Regulation of mRNA stability by proteins that bind AU-rich elements | 81 | 0.4965 | 1.5479 | 0.0082 | 0.0816 |
| Mitotic M-M/G1 phases | 166 | 0.4940 | 1.5430 | 0.0139 | 0.0838 |
| Interferon alpha/beta signaling | 61 | 0.9178 | 1.5352 | 0.0000 | 0.0883 |
| E2F mediated regulation of DNA replication | 33 | 0.5402 | 1.5313 | 0.0437 | 0.0897 |
| Meiotic recombination | 82 | 0.5220 | 1.5261 | 0.0552 | 0.0923 |
| Pyrimidine metabolism | 24 | 0.5809 | 1.5246 | 0.0180 | 0.0924 |
| APC-Cdc20 mediated degradation of Nek2A | 21 | 0.6161 | 1.5074 | 0.0448 | 0.1023 |
| RNA Polymerase I transcription | 84 | 0.5079 | 1.5081 | 0.0587 | 0.1032 |
| Complement cascade | 29 | 0.5944 | 1.5098 | 0.0317 | 0.1035 |
| Inhibition of the proteolytic activity of APC/C required for the onset of anaphase by mitotic spindle checkpoint components | 18 | 0.6449 | 1.5030 | 0.0390 | 0.1047 |
| Chromosome maintenance | 118 | 0.4348 | 1.5001 | 0.0253 | 0.1058 |
| Cell cycle, mitotic | 306 | 0.4436 | 1.4963 | 0.0157 | 0.1077 |
| Antigen processing: ubiquitination & proteasome degradation | 199 | 0.4416 | 1.4782 | 0.0122 | 0.1217 |
| Downstream signaling events of B cell receptor (BCR) | 92 | 0.4528 | 1.4793 | 0.0103 | 0.1222 |
| Phosphorylation of the APC/C | 17 | 0.5746 | 1.4719 | 0.0758 | 0.1263 |
| Kinesins | 24 | 0.4815 | 1.4676 | 0.0762 | 0.1288 |
| Apoptosis | 142 | 0.4194 | 1.4450 | 0.0102 | 0.1510 |
| Meiotic synapsis | 71 | 0.4871 | 1.4378 | 0.0845 | 0.1571 |
| RNA Polymerase I, RNA Polymerase III and mitochondrial transcription | 117 | 0.4236 | 1.4332 | 0.0397 | 0.1604 |
| Peptide ligand-binding receptors | 183 | 0.3567 | 1.4147 | 0.0279 | 0.1804 |
| Activation of genes by ATF4 | 24 | 0.5438 | 1.4092 | 0.0462 | 0.1854 |
| RNA Polymerase I promoter opening | 59 | 0.5406 | 1.3959 | 0.1502 | 0.2002 |
| PERK regulated gene expression | 27 | 0.5226 | 1.3768 | 0.0600 | 0.2245 |
| Signaling by Hippo | 20 | 0.4940 | 1.3695 | 0.1080 | 0.2319 |
| Amyloids | 79 | 0.4852 | 1.3650 | 0.1385 | 0.2356 |
| Growth hormone receptor signaling | 24 | 0.5372 | 1.3562 | 0.1243 | 0.2448 |
| Respiratory electron transport, ATP synthesis by chemiosmotic coupling, and heat production by uncoupling proteins | 80 | 0.6034 | 1.3530 | 0.1705 | 0.2464 |
| Packaging of telomere ends | 48 | 0.5284 | 1.3508 | 0.1485 | 0.2467 |
