# Supplementary figures and images for: A Transcriptional Signature of Fatigue Derived from Patients with Primary Sjögren’s Syndrome
Source: PLoS One. 2015 Dec 22;10(12):e0143970. doi: 10.1371/journal.pone.0143970 (PMC4687914; doi:10.1371/journal.pone.0143970)

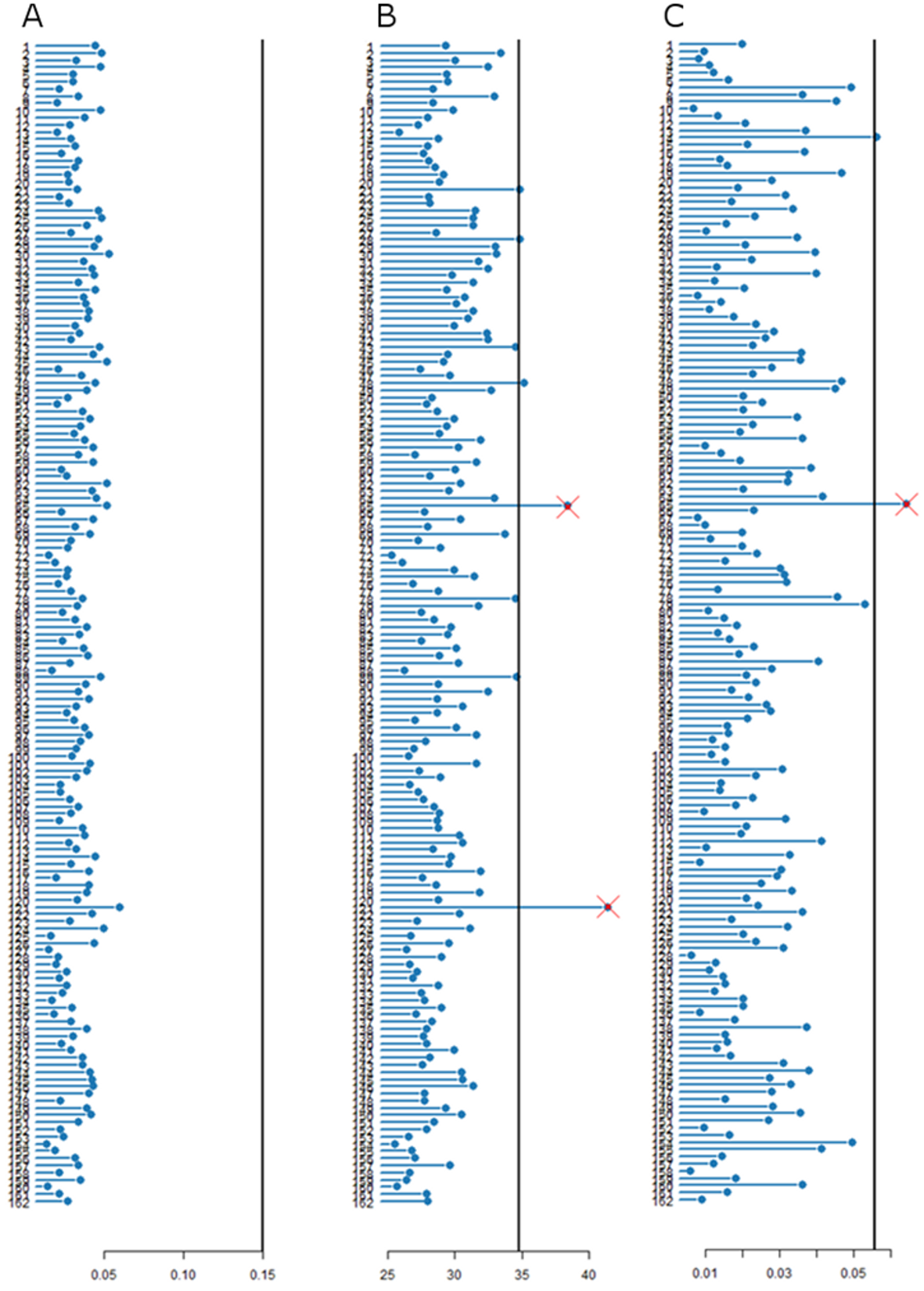

Supplement: S1 Fig — Bar charts of the three outlier detection methods. In each case the bars are shown in the original order of the arrays. Two arrays, numbers 61 and 121, were identified as outliers (red crosses). A) Hoeffding’s statistic D a. A threshold of 0.15 was used, which is indicated by the vertical line. No arrays exceeded the outlier threshold. B) The sum of distances to other arrays S a. Based on the distribution of the values across all arrays, a threshold of 34.8 was determined, which is indicated by the vertical line. Two arrays significantly exceeded the threshold and were considered outliers. C) The Kolmogorov-Smirnov statistic K a. Based on the distribution of the values across all arrays, a threshold of 0.0558 was determined, which is indicated by the vertical line. One array significantly exceeded this threshold and was considered an outlier. (PNG) [file pone.0143970.s005.png]

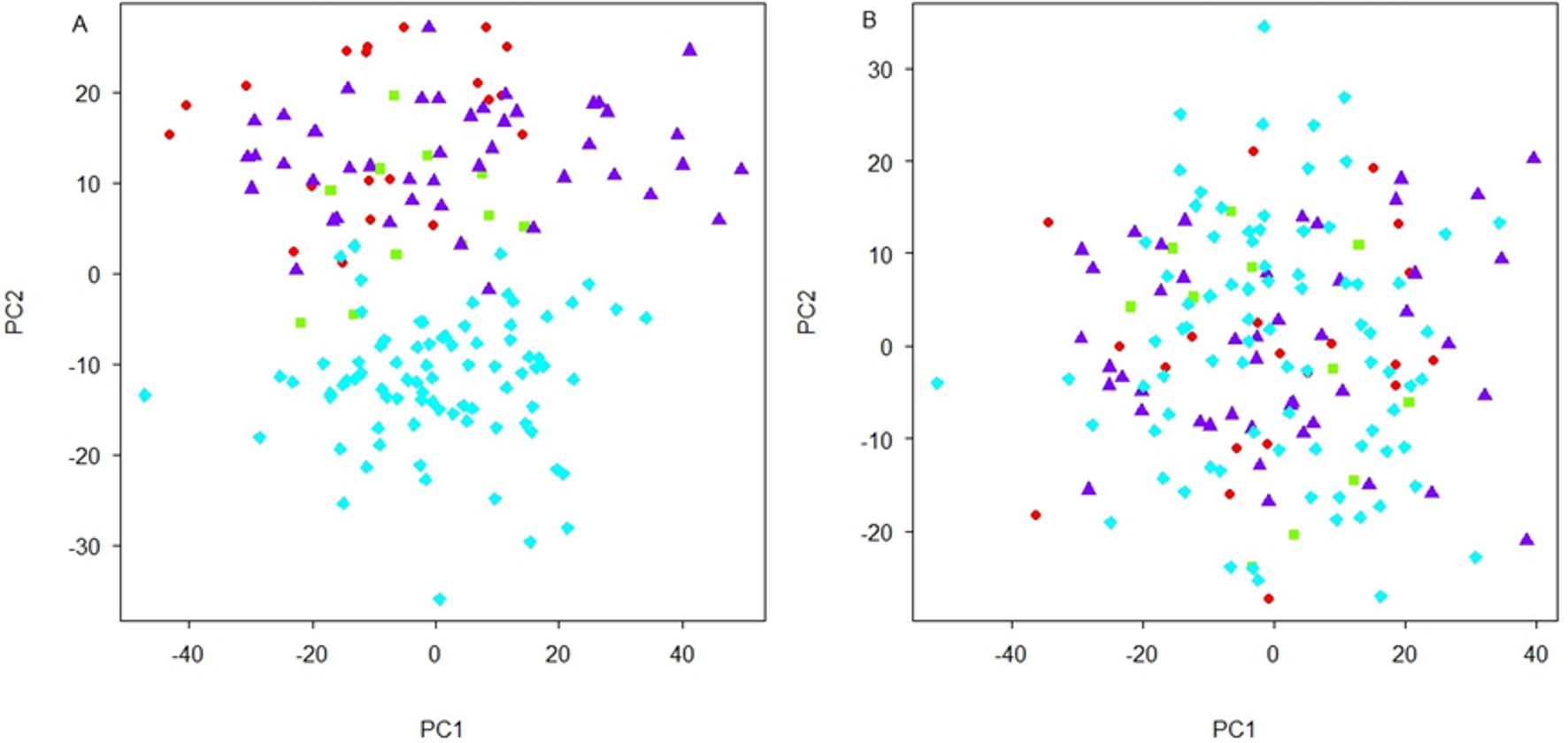

Supplement: S2 Fig — Principle component plots of the data pre- (A) and post-batch correction (B). Points are coloured and shaped by experimental batch. (PNG) [file pone.0143970.s006.png]

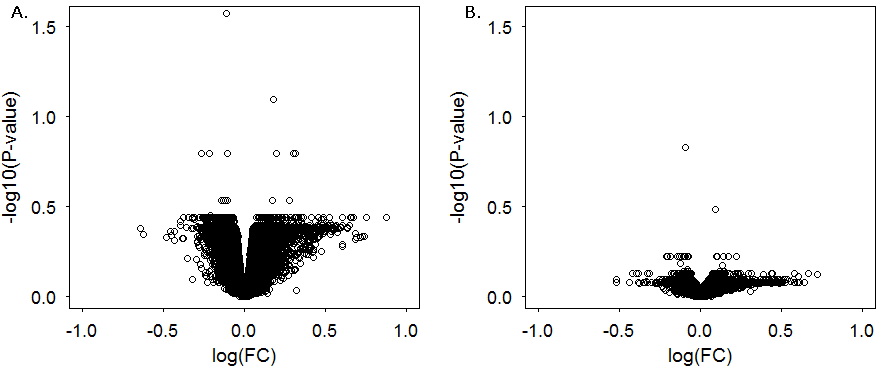

Supplement: S3 Fig — Volcano plots for fatigue groups using PROFAD and ESSPRI fatigue scores. The ranges of these scores are 0–7 for PROFAD and 0–10 for ESSPRI, respectively. No significantly differentially expressed genes were identified in either case. A. PROFAD, high fatigue >5 (n = 32) and low fatigue ≤2 (n = 32). B. ESSPRI, high fatigue >7 (n = 36) and low fatigue ≤3 (n = 34). (PNG) [file pone.0143970.s007.png]

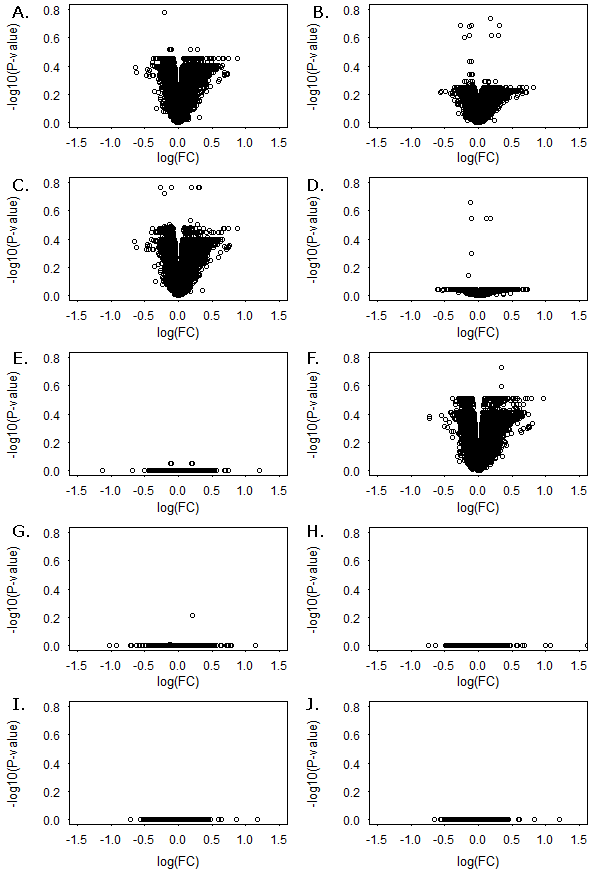

Supplement: S4 Fig — Volcano plots for the PROFAD fatigue groups corrected for clinical factors. High fatigue >5 (n = 32) and low fatigue ≤2 (n = 32). A. Age at UKPSSR cohort recruitment. B. Disease activity measured using the EULAR Sjögren’s Syndrome Disease Activity Index. C. Disease damage measured using the Sjögren’s Syndrome Disease Damage Index. D. The EULAR Sjögren’s Syndrome Patient Reported Index dryness sub-domain. E. The EULAR Sjögren’s Syndrome Patient Reported Index pain sub-domain. F. Anxiety measured using the Hospital Anxiety and Depression scale. G. Depression measured using the Hospital Anxiety and Depression scale. H. Pain and depression (E & G). I. Pain, depression, dryness and anxiety (D-G). J. All seven factors (A-G). No significantly differentially expressed genes were identified following any correction. (PNG) [file pone.0143970.s008.png]

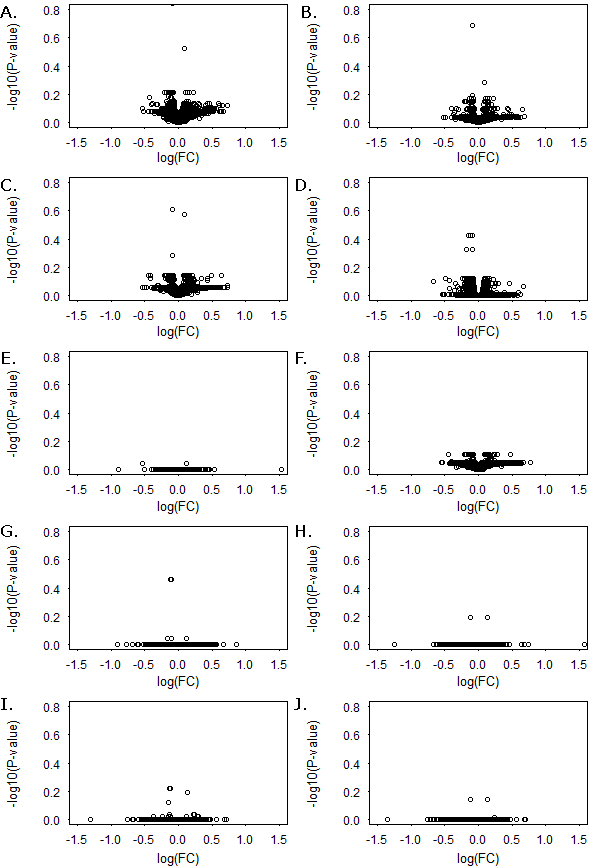

Supplement: S5 Fig — Volcano plots for the ESSPRI physical fatigue groups corrected for clinical factors. High fatigue >7 (n = 36) and low fatigue ≤3 (n = 34). A. Age at UKPSSR cohort recruitment. B. Disease activity measured using the EULAR Sjögren’s Syndrome Disease Activity Index. C. Disease damage measured using the Sjögren’s Syndrome Disease Damage Index. D. The EULAR Sjögren’s Syndrome Patient Reported Index dryness sub-domain. E. The EULAR Sjögren’s Syndrome Patient Reported Index pain sub-domain. F. Anxiety measured using the Hospital Anxiety and Depression scale. G. Depression measured using the Hospital Anxiety and Depression scale. H. Pain and depression (E & G). I. Pain, depression, dryness and anxiety (D-G). J. All seven factors (A-G). No significantly differentially expressed genes were identified following any correction. (PNG) [file pone.0143970.s009.png]

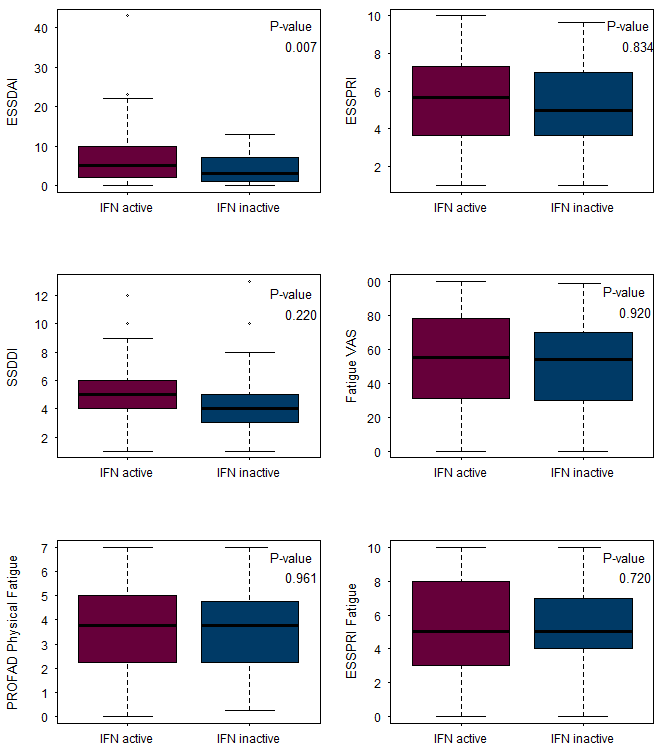

Supplement: S6 Fig — The clinical scores in the IFN type I positive and negative groups. ESSDAI scores were significantly higher in the IFN positive group. However, there was no significant relationship between IFN signature and ESSPRI, SSDDI or the three fatigue scores. (PNG) [file pone.0143970.s010.png]
